# Origin-Dependent Inverted-Repeat Amplification: Tests of a Model for Inverted DNA Amplification

**DOI:** 10.1371/journal.pgen.1005699

**Published:** 2015-12-23

**Authors:** Bonita J. Brewer, Celia Payen, Sara C. Di Rienzi, Megan M. Higgins, Giang Ong, Maitreya J. Dunham, M. K. Raghuraman

**Affiliations:** Department of Genome Sciences, University of Washington, Seattle, Washington, United States of America; Duke University, UNITED STATES

## Abstract

DNA replication errors are a major driver of evolution—from single nucleotide polymorphisms to large-scale copy number variations (CNVs). Here we test a specific replication-based model to explain the generation of interstitial, inverted triplications. While no genetic information is lost, the novel inversion junctions and increased copy number of the included sequences create the potential for adaptive phenotypes. The model—Origin-Dependent Inverted-Repeat Amplification (ODIRA)—proposes that a replication error at pre-existing short, interrupted, inverted repeats in genomic sequences generates an extrachromosomal, inverted dimeric, autonomously replicating intermediate; subsequent genomic integration of the dimer yields this class of CNV without loss of distal chromosomal sequences. We used a combination of *in vitro* and *in vivo* approaches to test the feasibility of the proposed replication error and its downstream consequences on chromosome structure in the yeast *Saccharomyces cerevisiae*. We show that the proposed replication error—the ligation of leading and lagging nascent strands to create “closed” forks—can occur *in vitro* at short, interrupted inverted repeats. The removal of molecules with two closed forks results in a hairpin-capped linear duplex that we show replicates *in vivo* to create an inverted, dimeric plasmid that subsequently integrates into the genome by homologous recombination, creating an inverted triplication. While other models have been proposed to explain inverted triplications and their derivatives, our model can also explain the generation of human, *de novo*, inverted amplicons that have a 2:1 mixture of sequences from both homologues of a single parent—a feature readily explained by a plasmid intermediate that arises from one homologue and integrates into the other homologue prior to meiosis. Our tests of key features of ODIRA lend support to this mechanism and suggest further avenues of enquiry to unravel the origins of interstitial, inverted CNVs pivotal in human health and evolution.

## Author Summary

Chromosomal aberration such as gene amplification is a common event in human diseases and is often selected during adaptation of microorganism to stress. We proposed a replication-based model to explain the formation of a particular type of genomic aberration: internal inverted DNA amplification with retention of the distal end of the chromosome. In this study, using yeast as a model, we test the feasibility of several of these steps for the formation of an inverted amplification: a specific DNA replication anomaly (1) leading to the formation of a palindromic extrachromosomal circular molecule (2) followed by the homologous reintegration of this molecule into the genome (3). A significant feature of this mode of amplification is that the amplified sequences contain one or more replication origins. The instability of the inverted junctions can lead, through homology driven processes, to more complex genomic structures that contain a partial triplication within a duplicated segment, a structure commonly found associated with human disease.

## Introduction

Eukaryotic genomes are surprisingly plastic. Inversions, deletions, and duplications of genetic material are commonplace in disease and evolution, arising through both homologous and non-homologous recombinational events and by errors in replication. Intrigued by the structure of a sequenced amplicon containing the *SUL1* locus in *Saccharomyces cerevisiae* [1], we proposed a novel mechanism for its production [2]. The inverted nature of the triplication (the central copy is inverted with respect to the two flanking copies), the sequences of the junctions, and the inclusion of the adjacent replication origin, *ARS228*, suggested to us a role for a specific replication error in the generation of this type of amplicon. Perusal of the literature of human inverted triplication added additional support to our proposed mechanism, as results from some human *de novo* triplications suggested that an extrachromosomal intermediate is involved in the generation of these syndrome-associated amplification events (e.g., [3]; reviewed in [2]).

**Fig 1:**
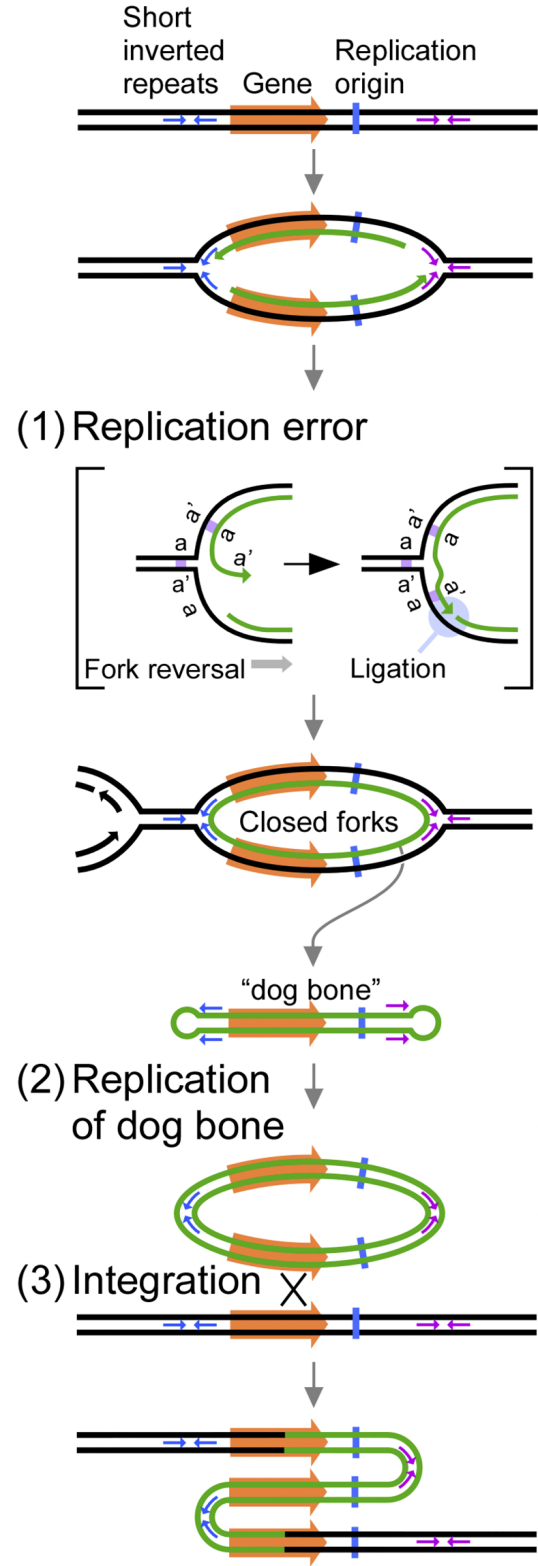
Key steps in the origin-dependent inverted-repeat amplification model for the generation of interstitial, inverted triplications. (1) Replication forks initiated at an origin of replication (vertical blue line) undergo fork reversal at small, interrupted inverted repeats (small blue/purple arrows; a and a’) leading to a replication error that covalently links nascent leading and lagging strands. These self-complementary DNA loops are expelled by adjacent replication forks to generate free linear DNA duplexes with closed loops (“dog bone” molecules) that correspond to the interrupted, inverted repeats and their intervening sequences. (2) In the next cell cycle, replication from the origin on the linear fragment generates a circular plasmid with the two copies of the amplified region in inverted orientation with the interrupted, inverted repeats at the head-to-head and tail-to-tail junctions. (3) Integration of the plasmid into the chromosomal locus generates the triplication with the central repeat in reverse orientation. The drawings are adapted from Fig 2 in [2].

We titled the mechanism ODIRA, for origin-dependent inverted-repeat amplification, because the yeast genomic region that is amplified includes an origin of replication, and because the head-to head and tail-to-tail junctions preserve the sequences of small, interrupted, inverted repeats that occur naturally in the genome [2]. The details of the model include the following steps (Fig 1): An error in replication fork progression causes ligation of the leading strand to the lagging strand at a replication fork that has regressed at the position of small, interrupted, inverted repeats. Because this fork is incapable of resuming replication we refer to it as a “closed” fork. If the two fork diverging from an origin of replication suffer the same error, a single stranded, closed loop results. (See [2] for outcomes that include a single closed fork.) An incoming fork from an adjacent replicon is expected to replicate through this region, displacing the closed loop by branch migration. The closed loop, being self complementary, forms a linear, double-stranded DNA (dsDNA) fragment that contains hairpin ends derived from the chromosomal inverted repeats. We refer to this structure as a “dog bone”. Since the dog bone was produced by errors at diverging forks, the dog bone will also contain an origin of replication. In the next cell cycle the dog bone can undergo replication to form an inverted dimeric plasmid. If the amplified sequences contain genes that are beneficial, cells with this self-replicating plasmid will enjoy a selective advantage. The plasmid can also integrate by homologous recombination into the original locus, producing an inverted triplication without loss of distal chromosomal sequences.

Since the publication of our model, we have collected data on additional amplification events that include *SUL1* in yeast. All of the events are interstitial triplications (or multiples of 2n + 1); each has an amplification of a unique, internal segment of chromosome II but nevertheless retains the distal portion of the chromosome; all contain the adjacent origin of replication, *ARS228*; and with one exception, all are bounded by junction sequences derived from chromosomally derived, short, interrupted, inverted repeats [4].

ODIRA is not the only model that attempts to explain the generation of inverted triplications. Generation of inverted amplification events has long been thought to be the consequence of chromosome breakage followed by replication of the broken chromosome and ligation of the centromere proximal ends to create a bridge at mitosis that is subsequently re-broken at mitosis (the Breakage-Fusion-Bridge or BFB cycle of [5]). However, certain features of the BFB cycle are not compatible with the observed structure of the triplication of the yeast *SUL1* locus in yeast: (1) in this haploid strain, there is no loss of sequences distal to the amplification breakpoints as expected for BFB cycles; and (2) the *SUL1* amplicons include uneven numbers of repeats (3, 5, 7, etc.)—BFB produces copy number expansion in equal integers (2, 4, etc.).

At least two other general models involving replication errors have been proposed to explain inverted triplications. One class, including MMBIR (Microhomology Mediated Break Induced Replication) and FoSTeS (Fork Stalling and Template Switching; reviewed in [6]), proposes that nascent strands at replication forks are promiscuous and highly mobile, moving around the genome in search of microhomology to continue their polymerization. A second class of model suggests that a small inverted repeat near a single-stranded nick can fold back on itself and prime synthesis, thereby peeling back the nicked strand to generate a palindromic hairpin [7]. At a second inverted repeat, the polymerase returns to the unbroken strand and fills in the gap. Both of these models have appealing features and on paper appear to be plausible; however, to our knowledge, there have been no attempts at recreating the stages of either replication error required for these models *in vitro* or *in vivo*.

In this work we have used a combination of *in vitro* and *in vivo* experiments to test some of the predicted steps in ODIRA (Fig 1). We find that (1) ligation of the leading nascent strand at a replication fork to the lagging nascent strand at a short, interrupted, inverted repeat can occur *in vitro*, (2) replication of the hairpin-capped linear (dog bone) efficiently generates the expected, circular, inverted, dimeric plasmid intermediate *in vivo*, (3) long term growth selects for cells in which integration of the dimeric plasmid into the genome has occurred and produces the expected inverted triplication; and (4) deletion of the origin next to *SUL1* alters both the size and structure of the recovered amplicons. In addition, through sequence analysis of two exceptional *SUL1* amplified clones we find potential secondary rearrangements of the inverted junctions. These two rearrangements are remarkably similar to a common form of human CNV that contains a partial inverted triplication lying within a larger duplicated segment (DUP-TRP/INV-DUP; [8]).

## Results

### Creating a closed fork *in vitro*

The central feature of the ODIRA model for gene amplification is an error at a replication fork where the leading strand 3’-end becomes ligated to the 5’-end of the nascent lagging strand. We reasoned that the most probable time for this ligation event to occur would be at a stage of replication fork progression where the leading strand polymerase has completed the synthesis of a pair of short inverted repeats but the inverted repeats on the lagging strand template lie in the Okazaki gap (Fig 1, brackets). Fork regression (reviewed in [9–11])—reannealing of the two parental strands with displacement of the newly synthesized leading strand—would provide an opportunity for the free leading strand to search for a pairing partner on the nearby single-stranded lagging strand template. *In vivo*, forks can regress to the point that the two nascent strands reform a fourth duplex at the fork (referred to as a “chicken foot” structure) but they frequently occur under conditions of genomic stress [9, 11, 12].

**Fig 2:**
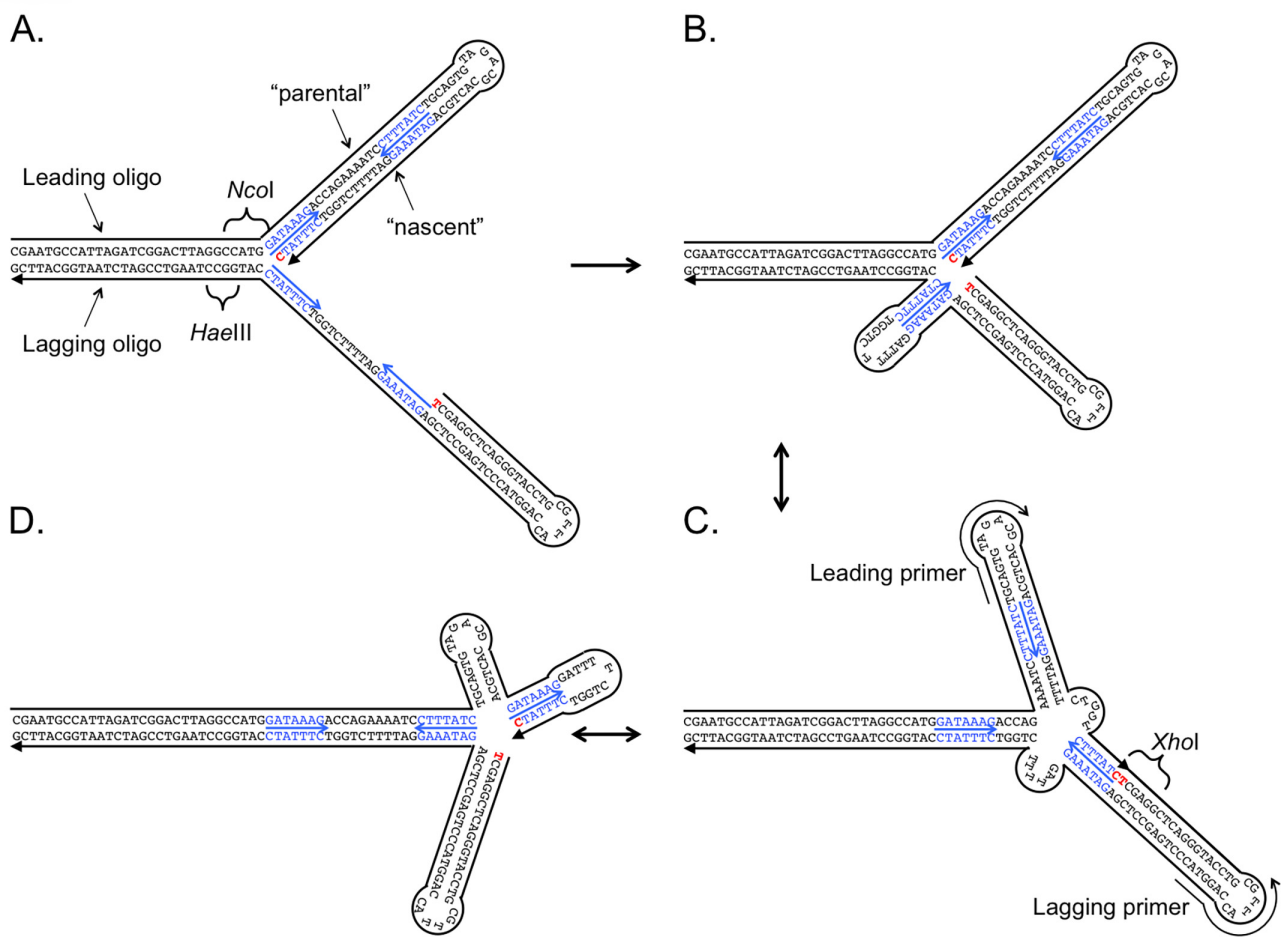
An *in vitro* construct to test for fork reversal and cross-strand ligation. An artificial replication fork was generated using commercially synthesized oligonucleotides (sizes of 100 and 99 nucleotides, with a 5’-phosphate on the 99 nucleotide lagging oligonucleotide). (A) To facilitate assembly of the fork from its component strands, the “parental” and “nascent” strands of the leading arm of the duplex, and likewise, the two strands of the lagging arm of the replication fork were covalently joined by a short linker. Complementary sequences in the two, 30 base, single stranded tails allow the fork to assemble *in vitro*. Features engineered into the two oligos include a 7 bp inverted repeat (blue sequences highlighted with small arrows) separated by 11 nucleotides of non-complementary sequence, and various restriction sites to facilitate evaluation of the fork and its products. (B) The single stranded stretch on the lagging strand exposes two complementary copies of the inverted repeat (in blue with arrow) which are expected to fold into a stem-loop structure to achieve the lowest free energy state for the forked molecule. (C and D) Breathing of the duplexes at the fork facilitates fork reversal and generates alternative conformations by branch migration—all with identical numbers of paired bases. The structures in B and D reflect the two extreme branch-migrated forms, with C representing only one of 25 possible intermediates. The two nucleotides in red are brought into proximity in the branch-migrated forms, providing a substrate for ligation by DNA ligase. Completion of the ligation step creates an *Xho*I restriction site. PCR primers (shown in C) target the two linkers.

To test whether such a nascent strand migration is possible across a replication fork, we created an artificial fork with this precise structure by annealing specifically designed oligonucleotides and tested for their ability to become ligated *in vitro* (Fig 2). We considered creating a forked structure by separately annealing two oligos for the leading strand template and its nascent leading strand and two oligos for the lagging strand template and its nascent strand, and then combining the two partial duplexes to form the unreplicated duplex portion of the fork. However, we feared that during annealing of the single stranded portions of the partial duplexes we would risk losing the nascent leading and lagging strands by branch migration even before we could set up the ligation reaction. To circumvent this potential problem we decided to link each pair of parental and nascent strands through a linker, creating a loop at their distal ends (Fig 2A). For the leading and lagging oligos (100 and 99 bases, respectively; see sequence in S1 Table), the duplex portions are present as a palindrome and the recreation of these duplex portions (after denaturation by boiling) occurs through a first-order reaction. Annealing the leading and lagging parental strands can then occur as a second order reaction without fear of losing the previously formed replicated duplexes to branch migration.

The other features we included in the design of the replication fork are several different restriction enzyme cleavage sites that allow us to confirm that we have created the desired structure (Fig 2A). In particular, we engineered a partial *Xho*I restriction site that encompasses the junction we hoped to create by ligation of the leading and lagging nascent strands (Fig 2C). If the 3’-C of the leading strand becomes ligated to the 5’-phosphorylated T of the lagging strand, an *Xho*I site is created that does not exist anywhere in any of the duplex segments of the fork. To test for the presence of the *Xho*I site after ligation, we designed PCR primers whose 3’-ends lie within the linkers connecting the parental and replicated strands with their 3’-ends directed towards each other across the fork (Fig 2C). Only if ligation is successful will we detect a PCR product, and that PCR product will be cleavable by *Xho*I.

**Fig 3:**
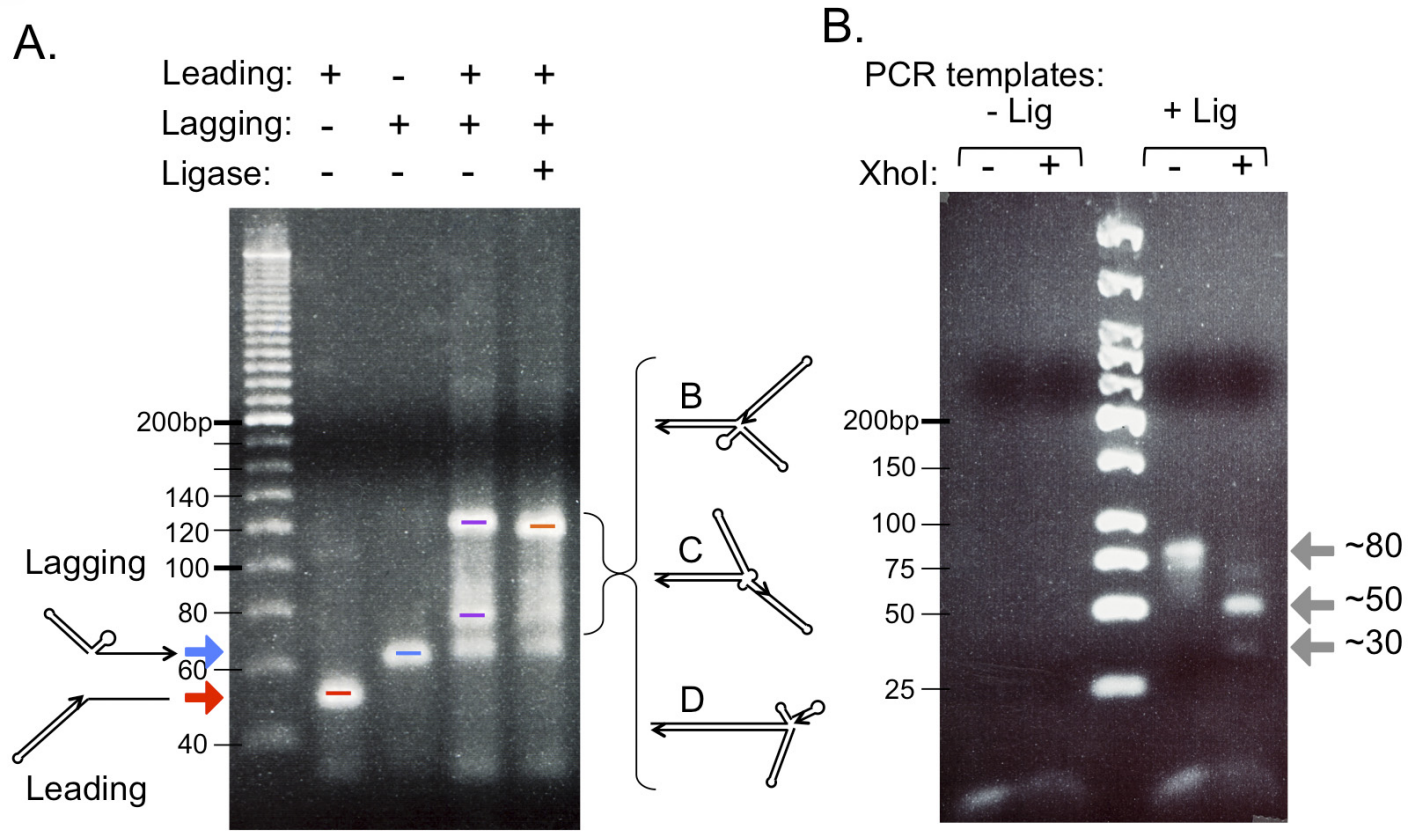
Ligation of leading and lagging “nascent” strands at a replication fork *in vitro*. (A) Each oligonucleotide was separately analyzed by electrophoresis in a 3.75% agarose gel after boiling and chilling on ice to allow the hairpins on both oligos to fold (leading strand marked with red arrow, lagging strand marked with blue arrow). The differences in migration of these nearly identically sized oligonucleotides are likely due to their different three-dimensional structures. The two oligonucleotides were mixed (in nearly equal molar amounts), boiled, chilled on ice and then returned to room temperature to allow the two complementary, single stranded tails to anneal. The image of the ethidium bromide stained gel reveals that some unreacted lagging strands remain (blue bar), but the majority of the oligonucleotides have formed higher molecular weight molecules (extremes highlighted with purple bars; intermediates from Fig 2 are labeled B, C, and D). The differences in migration of these molecules with identical mass are likely due to different branching patterns. Addition of DNA Ligase + ATP (last lane on the gel) results in a slightly faster migration of the slowest migrating form (orange bar) relative to its counterpart in the no-ligase lane. (B) Reserved samples from the last two lanes in (A) were used as templates in PCR reactions to detect a covalent linkage between the two oligonucleotides across the fork. PCR primers were designed such that their 3’ends resided in the single stranded linkers at the distal arms of the leading and lagging strands (see Fig 2C). The image of the ethidium bromide stained 3.75% agarose gel reveals a PCR product of 78 bp that can be cleaved by *Xho*I into 47 and 31 bp fragments (gray arrows).

To begin the annealing reaction with uniform structures we diluted the oligos, boiled and chilled them on ice (denaturation/snap-back) before examining them by agarose gel electrophoresis (Fig 3A). Each oligo was present as a uniform species; however, they differed in mobility even though they had nearly identical masses. It is difficult to confirm the actual size of these oligos using dsDNA markers because the oligos are expected to be partial duplex with single stranded tails. In addition, the lagging strand oligo has the potential of generating a stem loop structure from the inverted repeats in the single-stranded Okazaki gap, creating a multiply branched duplex that would affect its migration in agarose gels [13]. When we mixed the two oligos and carried out the denaturation/snap-back treatment, additional slower migrating species were formed (Fig 3A) ranging in apparent size from 80 to 120 bp. As there are 25 bases of homology over which branch migration can occur, we expect that many different structural isomers would be possible (Fig 2 illustrates three, with the two extremes in Fig 2B and 2D; S1 Movie) and that they would be dynamically interconverted. Addition of T4 DNA ligase modestly changed the distribution of structural forms with a slight increase in mobility of the most prominent form at ~120 bp (marked in orange, Fig 3A).

To determine whether ligation had joined the leading and lagging strands at the expected CpT junction, we used the annealed leading and lagging oligos, with and without ligase addition, as a template for PCR using the primers specific to the linker loops. In the absence of ligase, we detected no PCR products (Fig 3B). However, in the presence of ligase, we obtained a prominent PCR product of ~80 bp (expected size = 78 bp) that was cleavable by *Xho*I to generate 47 bp and 31 bp fragments (Fig 3B). (The 31 bp duplex fragment is probably unstable at the 37°C incubation temperature of *Xho*I and therefore not as prominently visible on the gel.) To confirm the identity of the PCR products, we treated the PCR product with S1 nuclease (which would cleave any single stranded portions of the 78 bp duplex) or with *Hae*III or *Nco*I (both sites present in the unreplicated portion of the forked molecule) and saw no alteration in the size of the PCR product (S1 Fig). From these results we conclude that a replication fork that contains short interrupted inverted repeats is capable of branch migration that facilitates ligation of the leading strand 3’-end to the 5’-end of the nascent lagging strand recapitulating the important features of step 1 of the ODIRA model, at least *in vitro*.

### Yeast can convert an artificial “dog bone” molecule into an inverted dimeric plasmid

After generating closed forks at both ends of advancing bidirectional forks, the nascent strands constitute a closed, self-complementary loop bounded by the inverted repeats that precipitated the generation of the closed forks (Fig 1). The same branch migration that created the closed fork would also be expected to expel the closed loop from the chromatid, especially in advance of an incoming fork that generates positive supercoils ahead of the fork (as described in detail in [2]). The positive supercoils could be relieved by promoting extrusion of the circular loop and allowing the parental strands to reform a duplex. During expulsion of the closed loop, the linked nascent strands would reform a perfect duplex ending in single stranded loops that originally resided between the interrupted inverted repeats (Fig 1-step 2). We have termed these predicted intermediates “dog bones”.

**Fig 4:**
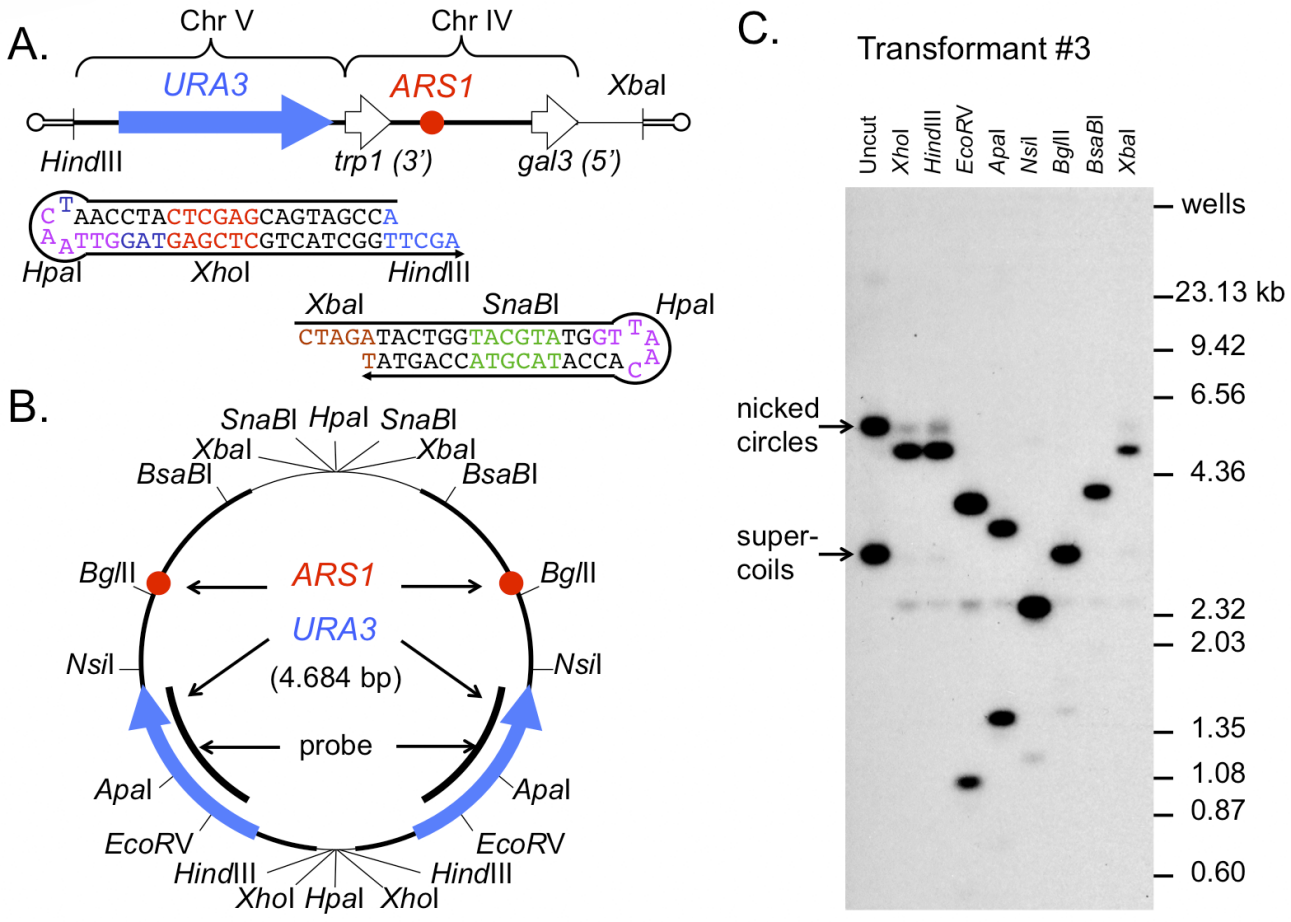
Transformation of yeast with an artificial dog bone results in inverted dimeric plasmids. (A) A *Hind*III-*Xba*I restriction fragment from plasmid pUA-DirB contains the *URA3* gene from chromosome V (blue arrow) and the origin *ARS1* from chromosome IV (red circle). Oligonucleotide hairpins were designed with *Hind*III (blue) and *Xba*I (brown) overhangs, 5’-phosphates to ensure their ligation to the ends of the *URA3-ARS1* fragment, and additional restriction sites in the duplex and loop portions to facilitate analysis of the DNA after transformation. (B) Map of the anticipated inverted dimeric plasmid after transformation and replication in yeast. Cleavage sites are indicated in bp. (C) Eight transformants were chosen at random; all eight had plasmids and five (transformants 1, 3, 5, 7, and 8) had restriction maps that were consistent with the map expected for a circular inverted dimer. The Southern blot using a *URA3* probe for transformant #3 is shown.

While others have created hairpin-capped linears and have shown that they can be maintained as plasmids in yeast [14, 15], we decided to create a dog bone with features that would allow us to easily follow its fate, both after transformation and during subsequent integration. We generated an artificial molecule from a *Hind*III/*Xba*I restriction fragment from a plasmid that had the *URA3* gene cloned upstream to a fragment that contains *ARS1* (plasmid pUA-DirB) and to which we ligated artificial hairpins that included several restriction enzyme sites in the stems and loops (Figs 4A and S2). Transformation of the *ura3Δ* strain BY4741 with the ligation product was very efficient (hundreds of transformants per μg of ligation product). We chose eight random clones for molecular analysis. If replication of the plasmid proceeded from *ARS1* we expected that the transformants would contain an inverted dimeric plasmid with the artificial hairpins, now in duplex form, at the head-to-head and tail-to-tail junctions (Fig 4B). We analyzed all eight transformants by restriction digests and Southern blotting and found that each contained *URA3*-*ARS1* plasmids. Two transformants contained a circularized version of the original 2.3 kb *Hind*III/*Xba*I fragment, and one contained a tandem, direct dimer of this fragment (produced through repair and ligation of un-capped fragments after transformation); these transformants were not investigated further. The five remaining clones each contained a circular plasmid of the anticipated size (~4.6 kb). To determine the structures of the plasmids, we carried out restriction digestion, gel electrophoresis and Southern blot hybridization using a *URA3* probe. The five, ~4.6 kb plasmids had the expected restriction sites (Fig 4 shows the Southern data for transformant #3). These results are consistent with previous reports of others [14] and support our expectation that a linear dog bone structure can be converted to a dimeric inverted plasmid through replication from the origin contained within the linear fragment that has ends protected by hairpin structures.

### Integration of the inverted dimeric plasmid into chromosome IV by homologous recombination

**Fig 5:**
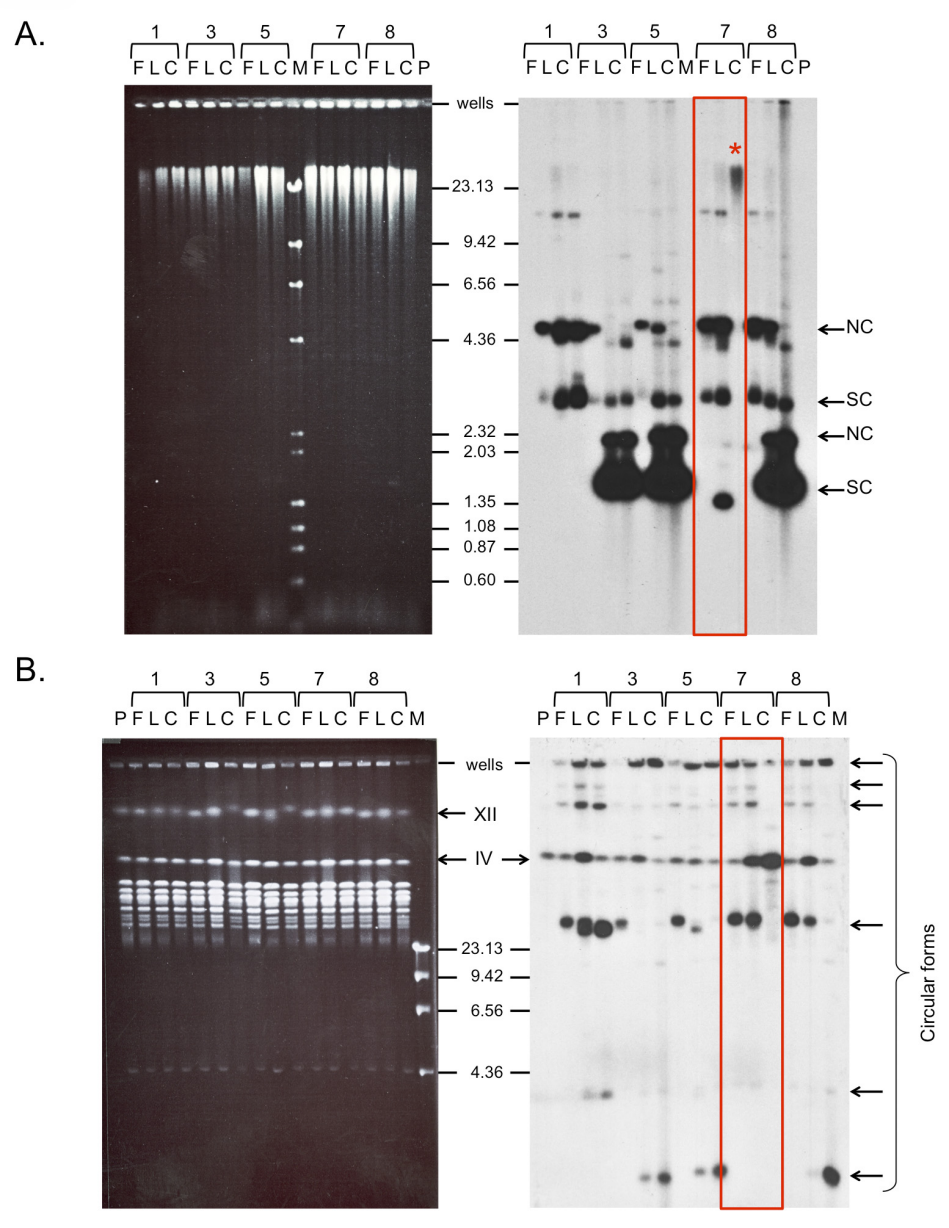
Long-term growth in selective medium selects for integration of plasmid sequences. After 80 to 90 generations of selective growth in–uracil medium, single clones were isolated from the cultures of transformants 1, 3, 5, 7, and 8. DNA from the first day (F), the last day (L) and the clone (C) was isolated by smash-and-grab and by an agarose plug method and analyzed by conventional (A) and CHEF (B) gel electrophoresis. The fates of the transforming *URA3-ARS1* dog bones were determined by Southern blots using *URA3* as a probe (ethidium bromide images on left, Southern hybridization on right). The parental strain BY4741 (P) has a complete deletion of *URA3* on chromosome V. (A) Plasmids were identified in all samples (nicked circular (NC) and supercoiled (SC) molecules of two different sized plasmids), with the exception of the clone from transformant #7 (red box). In this sample, hybridization to chromosomal fragments suggested that the plasmid had integrated (marked with *). (B) CHEF analysis confirms the presence of *URA3* on chromosome IV. Increased hybridization of *URA3* sequences (over the background signal found in the parental strain (P)) on the last days of selective growth suggests that integration had occurred in other cultures, although the single clones did not reflect this event. (Note that small plasmids migrate very slowly under CHEF gel electrophoresis conditions and also are trapped in the wells. These molecules are indicated by brackets on the right side of the gel.) Both DNA sampling techniques also revealed that free plasmids were experiencing other changes as a consequence of long-term selective growth.

The third step of the ODIRA model presented in Fig 1 proposes that the inverted, dimeric plasmid integrates into the genome to produce the inverted triplication. Upon initial characterization of dog bone transformants, we did not find any clones in which the *URA3* sequences had integrated, either through homology or by non-homologous means, into chromosomal DNA. To provide additional time for this event to occur we performed batch culture evolution experiments in which we serially diluted each of the five clones containing the ~4.6 kb plasmid (clones #1, 3, 5, 7, and 8) 1000-fold every two days into fresh selective medium. The host strain we chose, BY4741, has a complete deletion of the *URA3* locus, with no complementarity to the sequences of *URA3* in our dog bone molecule. This choice of strain served two purposes. First, the lack of homology at the *URA3* locus prevented the recovery of uracil prototrophs through gene conversion of the chromosomal locus using dog bone sequences. Second, if the plasmid had integrated by homologous recombination into a chromosome, it would be restricted to use the homology in the region of *ARS1* on chromosome IV. Samples from the serial batch cultures were collected at each dilution for analysis. On the last day (transfer 8 for some cultures and transfer 9 for others) cells were streaked onto uracil-dropout (C-uracil) plates and a single clone was picked from each population. We isolated DNA from population samples grown in liquid C-uracil medium and harvested on the first day (F), on the last day (L), and from the last-day clone (C) for analysis by conventional and CHEF gel electrophoresis. Analysis of uncut samples revealed the presence of plasmids (supercoiled and nicked forms) in all samples except for one—the last day clone from transformant #7 (Fig 5A right, Column C). This sample showed no evidence of free plasmid but had conspicuous hybridization of the *URA3* probe to the chromosomal fragments at the top of the gel, suggesting integration of the plasmid into the genome (Fig 5A, noted with an asterisk on the Southern blot; compare to Fig 5A left). To determine where in the genome this integration had occurred, we analyzed whole chromosomes from each sample by CHEF gel electrophoresis under conditions where we could unambiguously distinguish between integration into chromosome IV (the chromosome that contains *ARS1*) and chromosome XII (all other smaller chromosomes run in close proximity ahead of these two chromosomes; Fig 5B, left). While there is a small amount of cross hybridization of the *URA3* probe to unknown sequences on chromosome IV (Fig 5B right, lane “P”), there is a significant increase in signal for the last day clone from transformant #7 (Fig 5B right, lane C for transformant #7). None of the other last day random clones we selected had integrated the *URA3* sequence into their genomes. However, it was suggestive, from the hybridization signal over chromosome IV on the last day samples, that the other cultures, similar to the last day sample from transformant #7, also had produced integration events that were present in the population but not in the clone we had selected.

**Fig 6:**
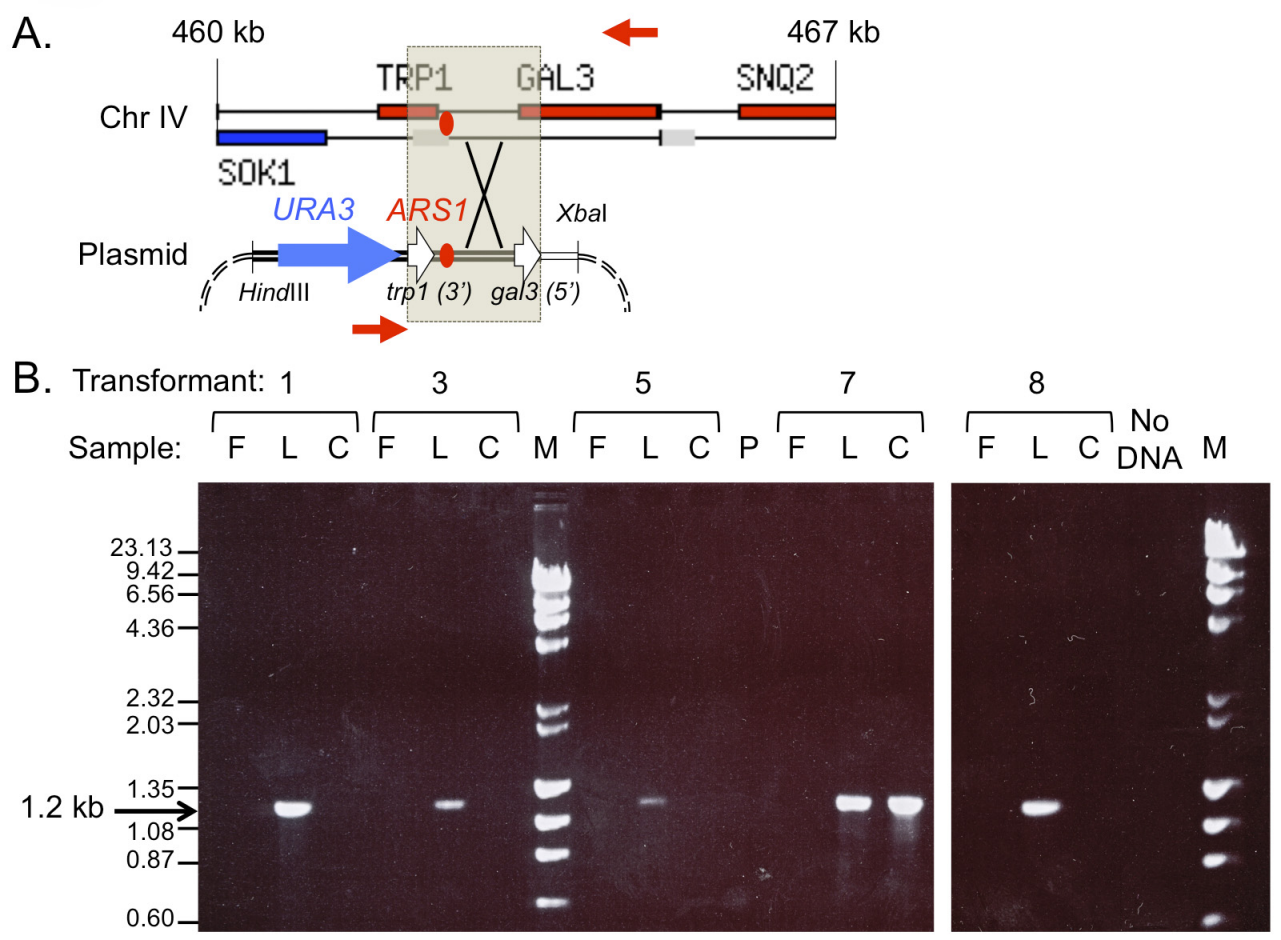
PCR amplification across the *URA3-GAL3* junction confirms plasmid integration adjacent to *ARS1* on chromosome IV. (A) The map illustrates the region of homology (shaded in grey) shared between chromosome IV (top; genomic region from *SOK1* to *SNQ2*—coordinates 460 to 467 kb—taken as a screen shot from the *Saccharomyces* genome database; www.yeastgenome.org) and the plasmid sequences (bottom). The position of PCR primers (red arrows) predicts a 1.2 kb fragment for integrants that have recombined by homology in the *TRP1*-*GAL3* region. (B) Ethidium bromide stained gel of PCR fragments. The presence of 1.2 kb PCR fragments in each of the last day samples (L) and clone (C) from the last day of transformant #7 confirms the integration site of the plasmid sequences.

To determine whether *URA3* sequences had integrated into chromosome IV in all of the evolution experiments, and whether they had integrated by a homology dependent mechanism, we designed PCR primers between the *URA3* sequences on the plasmid and the *GAL3* sequences on chromosome IV (Fig 6A, red arrows). If integration occurred by homologous recombination we expected a PCR fragment of 1.2 kb present only in the last day populations and in the clone from transformant #7. The gel results of PCR samples conducted on the three samples (first day, last day and clones) from each transformant confirm the existence of homology directed integration, in a subset of cells, by the last day of each of the evolution experiments (Fig 6B).

**Fig 7:**
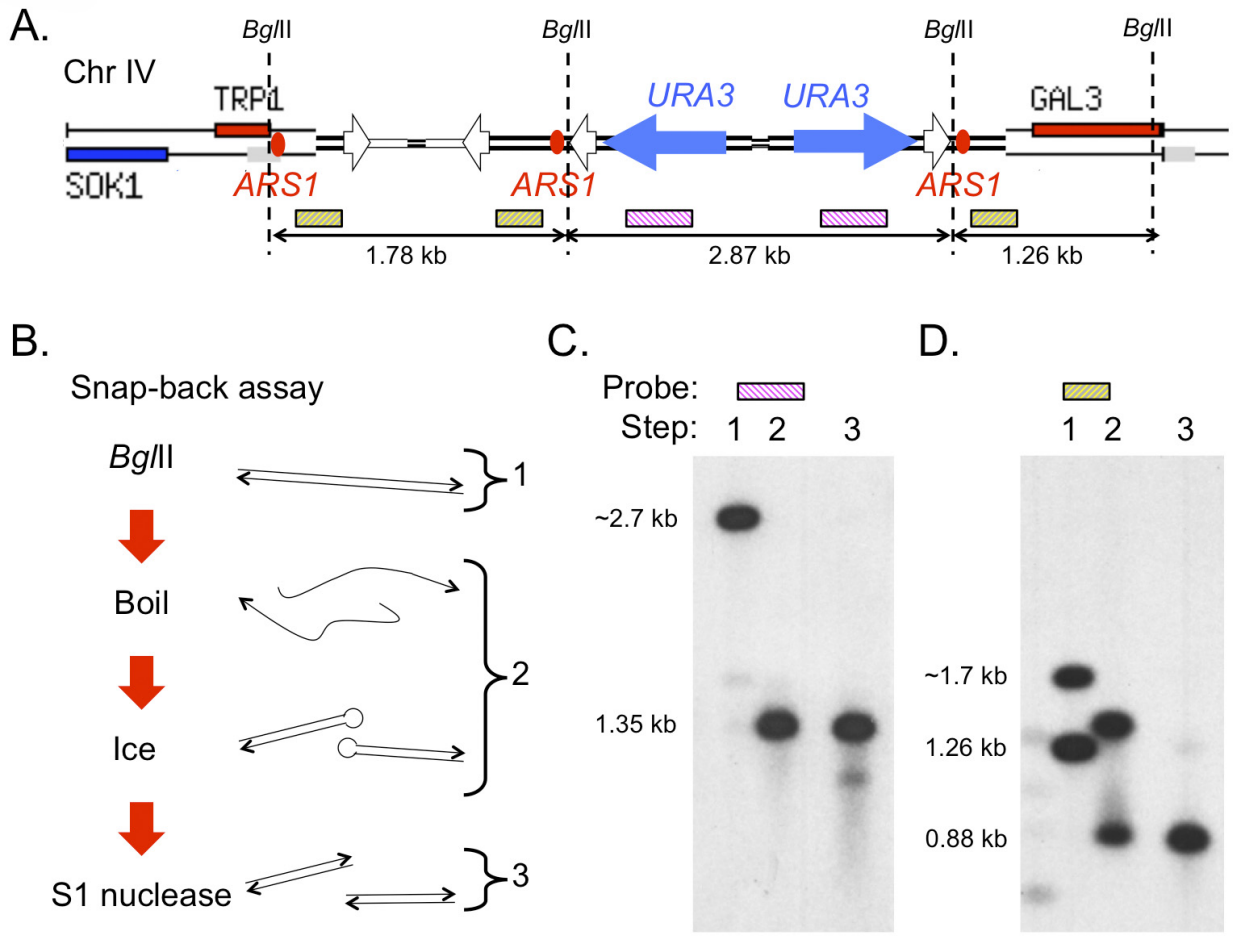
The inverted dimeric plasmid integration preserves both palindromic junctions, creating an inverted triplication of *ARS1*. (A) The map illustrates the expected structure after homologous integration of the dimeric plasmid between *TRP1* and *GAL3* on chromosome IV. The positions and sizes of *Bgl*II fragments, as well as probe sequences are illustrated. (B) A snap-back assay detects palindromic sequences by virtue of their ability to recreate duplex fragments of half the original size after boiling and quick cooling on ice. Additionally, these sequences are resistant to the ssDNA nuclease S1. (C and D) Southern blots of snap-back assays on the two palindromic junctions of the integrated plasmid in the last day clone from transformant #7. Lane 1 = native duplex *Bgl*II fragments; lane 2 = denatured/renatured palindromic duplexes; lane 3 = S1 nuclease resistant duplex DNA. The probe in (C) detects the *URA3* palindromic fragment; the probe in (D) detects the *ARS1* palindromic fragment as well as the non-inverted copy of *ARS1* adjacent to *GAL3*. Note that the larger ssDNA fragment (in lane 2) is degraded by S1 (lane 3).

As part of the ODIRA model, we propose that the dimeric inverted plasmid integrates directly into the chromosomes by homologous recombination to generate an inverted triplication [2]. However, given that we see both small and large changes in plasmid size over the short evolution experiments (Fig 5A and 5B right; and see below), we wanted to determine whether the entire, unrearranged plasmid had integrated into the genome, by determining whether each of the palindromic junctions in the dimeric inverted plasmid were also present in the clone from transformant #7. To detect these palindromic sequences, we digested the chromosomal DNA with *Bgl*II (Fig 7A) and performed snap-back assays (Fig 7B) using both *URA3* and *ARS1*-adjacent probes (Fig 7C and 7D). If a restriction fragment is palindromic, then after boiling and chilling on ice, it is expected to instantaneously reform a duplex that is half of the original molecular mass and be resistant to digestion by the single-stranded specific nuclease S1 (Fig 7B; [4]). We observed a single *URA3* fragment of ~2.7 kb that reformed as a 1.35 kb fragment in the snap-back assay and survived S1 treatment (Fig 7C). Similarly, the ~1.7 kb *ARS1* fragment reformed as a 0.88 kb fragment that also survived S1 treatment (Fig 7C). The 1.26 kb *ARS1* fragment provides an internal control: this fragment is not expected to contain a palindrome (see Fig 7A). Upon denaturation the single stranded version of this 1.26 kb fragment ran at a slightly retarded size (expected from the migration properties of a random coil of single stranded DNA) and was degraded by treatment with S1 (Fig 7D, lane 1 and 2). These results confirm that the dimeric, inverted plasmid, generated by *in vivo* replication of the artificial dog bone, is capable of homologous integration into yeast chromosomes to generate an inverted triplication.

### Rearrangement of palindromic junctions *in vivo*

As mentioned above, the inverted, dimeric plasmids derived from replication of the synthetic dog bone molecule appeared to be structurally unstable over the course of the ~100 generations of serial transfer in medium selecting for maintenance of the plasmid (Fig 5). Southern blot analysis of uncut plasmids on conventional and CHEF gels suggested that both small and large changes in plasmid size were occurring. To explore the nature of the diminished plasmid sizes, we performed restriction digests and Southern blots on the first day samples and the final clones for transformants 1 and 5, which showed modest and large alterations in plasmid size, respectively. The Southern blots (S3 Fig) revealed two types of rearrangements: loss of the restriction sites across the *URA3*-proximal hairpin (transformant #1) and complete deletion of one of the palindromic arms with retention of the restriction sites present in both hairpins (transformant #5).

**Fig 8:**
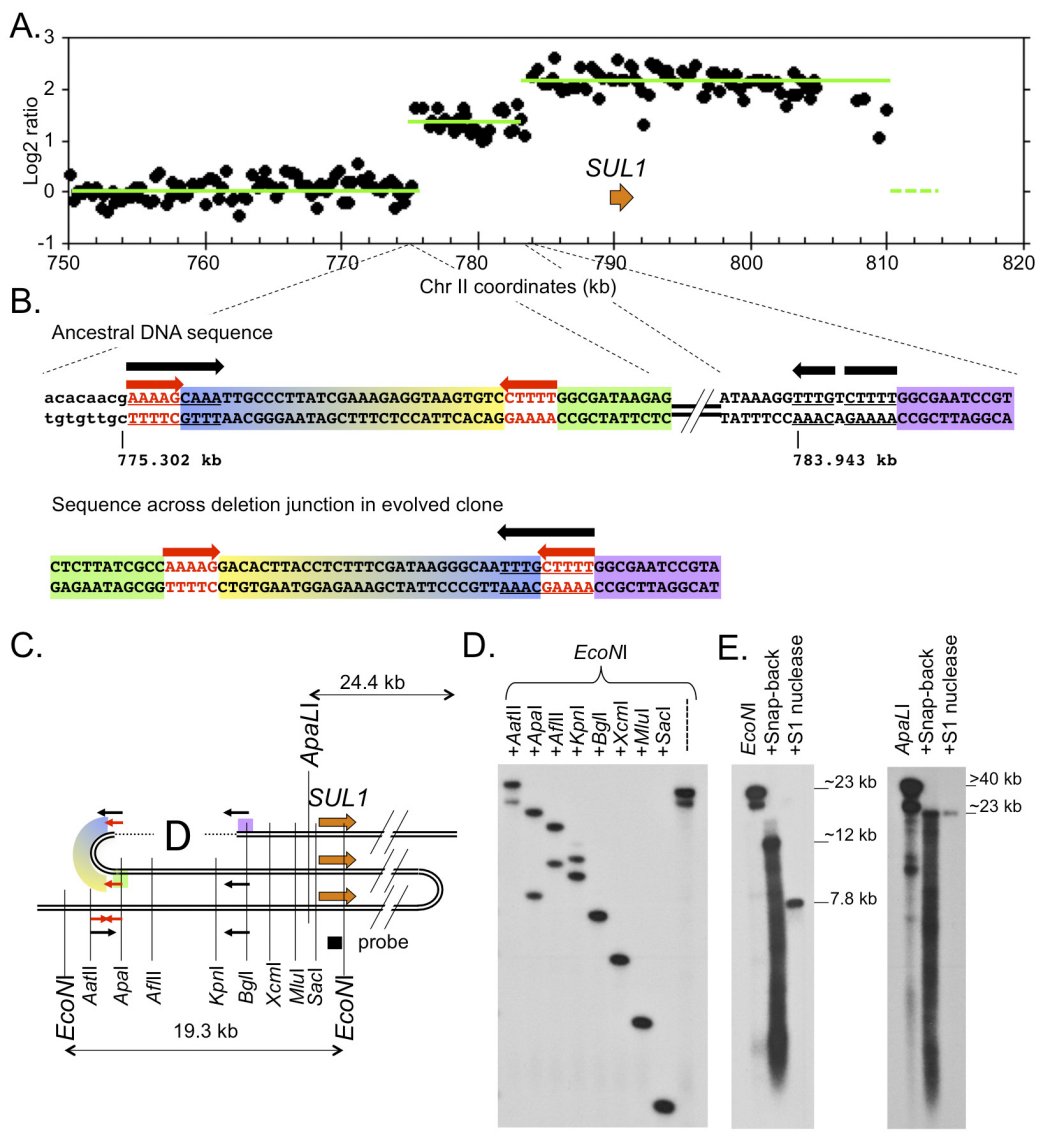
Secondary rearrangement at a palindromic junction in a *SUL1* amplicon. (A) ArrayCGH of yeast strain 10206-c1476. Only the last 65 kb of chromosome II are shown. (Due to the lack of unique probes near the right telomere, it is not possible to determine from aCGH the precise right junction of the amplicon.) (B) Sequences of the ancestral chromosome and the rearranged palindromic junction from the evolved clone. Sequences centromere-proximal of the inversion junction are in lower case. The short inverted repeats proposed to be the site of fork closure are indicated in red font (red arrow); sequences believed to be the sites of intramolecular, non-allelic, homologous recombination to generate the deletion of one arm of the palindrome are underlined (black arrow). Colored highlights are included to illustrate the orientation and junctions created after formation of the amplicon and the deletion of one of the palindromic arms. (C) Map illustrating the structure of the original inferred inverted triplication, highlighting the short inverted repeats (red arrows) and the sites of homology (black arrows). Colored blocks are the same as in (B). The direct orientation of the two distal black arrows creates an opportunity for intramolecular, non-allelic, homologous recombination to remove the intervening sequences. (D) Confirmation of the structure of the centromere-proximal amplicon junction by indirect end-labeling. DNA from 10206-c1476 was digested with *EcoN*I, distributed to 9 tubes and digested with one of the indicated enzymes. A probe (black square) adjacent to the *EcoN*I site detects fragments that extend from the *EcoN*I site toward the centromere. The map in (C) illustrates the locations of sites in the ancestral genome; these fragment sizes are detected for the most centromere proximal copy of the *SUL1* locus and for fragments completely contained within the two arms of the inverted amplicon. The second bands seen for *Apa*I, *Afl*II, and *Kpn*I are consistent with sizes expected for fragments that span the deletion. (E) Snap-back assays on *EcoN*I and ApaLI digested genomic DNA from 10206-c1476. Both ancestral and amplicon specific fragments are detected in native DNA. Denaturation and quick cooling produces a smear of ssDNA fragments along with duplex hairpins of approximately 12 kb for the *EcoN*I digest and 23 kb for the *ApaL*I digest. There is no change in size of the ApalI fragment after S1 nuclease digestion, but only a 7.8 kb duplex portion of the *EcoN*I hairpin remains after S1 digestion. The change in size is consistent with the *EcoN*I hairpin having lost a large single-stranded loop during S1 digestion.

The loss of one arm of the inverted, dimeric, *URA3-ARS1* plasmids is reminiscent of rare imperfect inverted triplications we had previously encountered amongst *SUL1* amplification events. Among the dozen naturally occurring *SUL1* amplicon structures that we previously examined in clones isolated after 200 generations of growth in a sulfate-limited chemostat [4, 16], the vast majority had stable palindromic junctions. However, we found two exceptions: array Comparative Genome Hybridization (aCGH) analysis of both of these clones suggested that the centromere proximal junctions were complex, with step-wise increases in copy number from 1 to 2 to 3 over the span of several kb (Figs 8A and S4A), reminiscent of the aCGH analysis of human DUP-TRP/INV-DUP amplicons [8]. Using split-read analysis [17] of the whole genome sequences from these two clones, we were able to determine the sequence across these complex centromere-proximal junctions (Figs 8B and S4B). Comparing sequences from the evolved clones with those of the ancestral genome, we propose that the initial amplification event occurred through the ODIRA mechanism, but subsequently, a deletion of one arm of the palindrome occurred by a homologous reaction between two short repeats—one at the initial inversion junction and the second at a distal site in the amplicon (Figs 8C and S4C). In the ancestral genome, these repeats are in inverted order; however, after creating an inverted triplication, two copies come to lie in direct orientation. While repair or replication errors could produce the precise deletion in one of the palindrome’s arms, it could also occur by intramolecular, non-allelic, homologous recombination.

To confirm the structures suggested by aCGH and genomic sequencing, we subjected DNA from these two clones to Southern blot analysis using both indirect end-labeling of double digests (Figs 8D and S4D) and snapback assays (Figs 8E and S4D). The restriction maps inferred for the amplicons are compatible with a deletion of one of the arms of the palindrome and the retention of the palindrome’s second arm and one copy of the ancestral sequence. The snap-back assays reveal a substantial change in size of the duplex after S1 treatment, consistent with a very large loop (spanning the deleted arm of the palindrome; Figs 8E and S4E). While we do not have the un-deleted amplicon for either of these clones, the pathway we suggest provides an alternate to FoSTeS for their generation and for the generation of DUP-TRP/INV-DUP amplicons in human cells.

### The role of *ARS228* in generating inverted *SUL1* amplicons

A key feature of the ODIRA model for producing interstitial inverted amplicons is that the segment of amplified DNA must contain one or more origins of replication. This condition follows from the hypothesis that to generate a free dog bone, two diverging forks would have to suffer the same closed-fork fate and can be met only if the intervening sequence contains one or more origins of replication. Since the origin *ARS228* lies immediately downstream of the *SUL1* gene, we tested the importance of *ARS228* in the process of *SUL1* amplification by replacing the *ARS228* ARS consensus sequence (ACS) with a scrambled, non-functional copy, *ars228Δ*, and repeated seven sulfate-limited chemostat experiments. We confirmed that *ars228Δ* eliminates origin function by analyzing chromosomal replication intermediates across the *ARS228* region by 2D gel electrophoresis [13] (S6 Fig).

**Fig 9:**
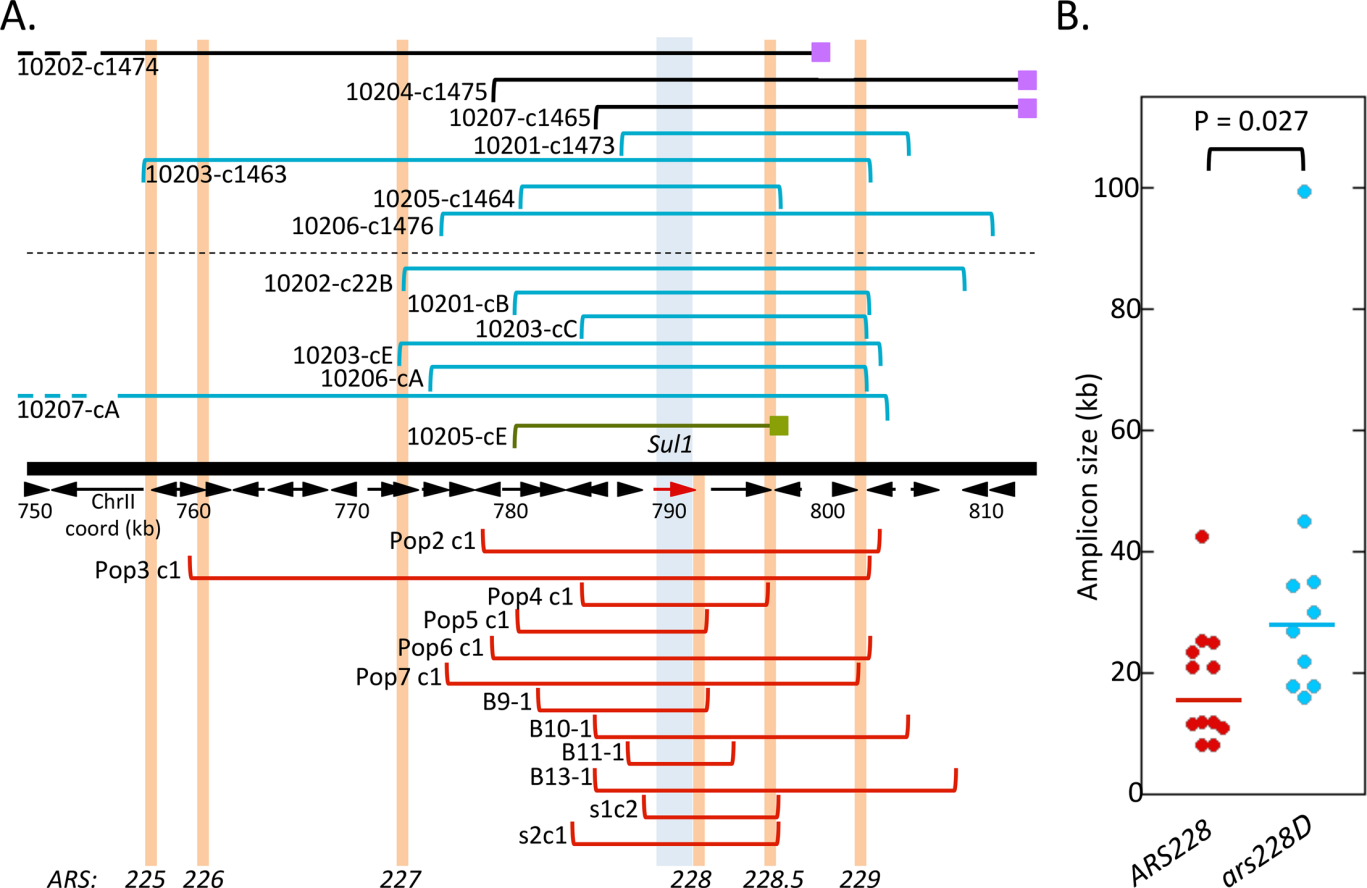
*SUL1* amplicon structures generated in the presence and absence of *ARS228*. (A) Top: Seven sulfate limited chemostats were grown for ~200 generations with a strain deleted for *ARS228*. A single random clone was isolated from each chemostat and characterized by *ApaL*I snap-back assays (S5 Fig) and aCGH (the amplification junctions are marked by brackets above the dotted line, aligned with the chromosomal map of the right arm of chromosome II from coordinates 750 kb to telomere (813 kb)). Among the single clones (S10201 to 7) we identified four clones with interstitial inverted amplification events (blue brackets) and three clones which were inconsistent with inverted amplification structures (black brackets; 10202-c1474 also suffered a telomere deletion). Additional unique clones (designated as cA-cE, below the dotted line) were analyzed from each chemostat culture. Among the seven additional clones, one clone was a complex isochromosome with deletion of one copy of *CEN2* (green clone). The junction sequence (green square) was not pursued further. (A) Bottom: Wild type cultures (*ARS228*) selected from the sulfate-limited chemostat cultures (~200 generations) were pooled from published work from our labs and their amplification junctions are shown below the chromosome map (red brackets). Molecular characterizations of chromosome II structures in these clones are consistent with interstitial inverted amplification. Pop 2–7 clones are from [4]; B9, 10, 11, and 13 are from [18]; and s1c2 and s2c1 are from [16]. The vertical orange bars mark the positions of known ARSs. (B) Sizes of amplified regions from the *ARS228* and *ars228Δ* strains generate medians of 16.5 and 28.9 kb, respectively, with a Wilcoxon Mann Whitney significance p-value of 0.027.

We isolated a single random clone from each of the seven *ars228Δ* cultures (Fig 9A top, above the dotted line) and found that four had an inverted, interstitial *SUL1* amplicon (S5 Fig, left). The remaining three had amplicons that extended to the right telomere (including one that had suffered a telomere deletion) and were not present as inverted copies (S5 Fig, right). The extra copies in these three latter clones are either found in direct orientation on the right arm of chromosome II or are translocated to telomeric regions of other chromosomes. To increase the sample size of inverted amplicons from the *ars228Δ* experiments, we screened additional clones, specifically looking for inverted amplicons (Fig 9A top, below the dotted line). From the aCGH we determined that one of these clones (10205-cE) carried an isochromosome with a deletion of one copy of *CEN2* and a deletion of sequences from the inversion junction to the right telomere. It was not characterized further.

The ten inverted clones from the *ars228Δ* experiments were compared with twelve clones culled from our previously published data in which the *ARS228* wild type culture was evolved in sulfate-limiting chemostats (Fig 9A bottom). While the numbers of evolved clone are small, there are three striking comparisons that we can make between the *ARS228* and *ars228Δ* amplicons. First, among the seven randomly chosen clones from the *ars228Δ* experiments three had a non-inverted amplicon structure. The *ARS228* strain uniformly produces inverted *SUL1* amplicons. These results suggest that without a functional copy of *ARS228*, an alternate mechanism of amplification has become more prominent. Second, in comparing the sizes of the amplified regions in those strains with an inverted amplicon, the mean sizes are different between the *ARS228* and *ars228Δ* strains—with median amplicon sizes of 16.5 and 28.9 kb, respectively (p = 0.027). Third, based on the aCGH data, it appears that all amplicons contain at least one potential origin of replication. However, several of the smallest amplicons from the two strains (Pop5-c1 and 10205-c1464) have hairpin junction adjacent to the single ARS contained on the amplified fragment. To determine whether these ARSs were in fact functional as origins in this new context, we conducted 2D gel analysis of the ARSs at the junctions: *ARS228* for Pop5-c1 (S7A and S7C Fig) and *ARS228*.*5* for clone 10205-c1464 (S7B and S7D Fig). Using restriction digests that generate different fragment sizes for the ARSs in the junction fragments compared to the same sequences in the telomere proximal ancestral fragment (S7E Fig), we were able to assess the origin function at the rearrangement junction. In both cases, these ARSs were functional origins (generating bubble structures; S7A and S7B Fig). We therefore conclude that each inverted amplicon contains at least one active origin of replication. When *ARS228* is deleted, all amplicons include the next, telomere-proximal origin, *ARS228*.*5*. In contrast, there is no apparent requirement to include *ARS228*.*5* in the amplified segment in strains with the functional *ARS228*. Taken together, these results support the “origin-dependence” tenet of the ODIRA mechanism. Furthermore, without a functional origin at *ARS228*, cells are more likely to use an alternate mechanism of gene amplification that does not result in inverted interstitial amplicons.

## Discussion

The most frequent and easily explained chromosomal amplification events occur by unequal sister chromatid exchange through homologous recombination at short directly repeated elements scattered throughout the genome. When these events occur intrachromosomally, they result in one chromatid that gains a copy of the segment between the direct repeats and the other chromatid that loses the segment. In haploids the loss of information could be lethal, but for the cell inheriting the duplication, there could be a selective advantage that favors its persistence in the population. The recombination event results in the extra copy being in direct orientation with respect to the original copy; the sequences distal to the duplication are retained. There are many examples in the literature of just this kind of amplification event (reviewed in [19]). The chromosomal domain that contains *SUL1* is unusual in that there are no directly repeated elements (no Ty retrotransposons, their long terminal repeats (LTRs) or tRNAs) that could easily generate direct repeats of a *SUL1* segment. The *SUL1* amplification events are overwhelmingly found as triplication or multiples of 2n+1 with the central copy inverted and with distal segments intact [1, 4].

The ODIRA model we have proposed can explain the formation of the *SUL1* amplicons by invoking a rather simple, but presumably rare, replication error that occurs when a replication fork progresses through closely spaced inverted repeats [2]. To produce the intermediate for interstitial inverted amplicons, two replication errors must occur at oppositely oriented forks—a requirement that would increase the rarity of dog bone formation. However, the two closed forks need not occur at forks proceeding from a single origin. It is also possible to create larger dog bones if the replication error occurs at any two diverging forks along a chromosome, regardless of the origin from which they arose. In fact, only four of the 22 inverted amplicons we have characterized in detail contained a single origin of replication—suggesting that forks from different replicons could have contributed to the inversion events. This latter situation is relevant when considering events in mammalian genomes, where replication origins are more broadly distributed across intergenic regions with an average spacing of 50 to 300 kb [20, 21] and where inverted amplicons, which are often identified by FISH patterns on karyotypes, can be hundreds of kilobases to several megabases long (see, for example, [22, 23]).

Single closed forks can also generate amplification events [2]. A centromere proximal closed fork would produce an acentric linear inverted chromosomal fragment, while a telomere proximal close fork would lead to isochromosome formation with deletion of telomeric sequences. In our limited set of clones we did not detect any acentric fragments, but clone 10205-cE, an isochromosome with a deletion of one of the copies of *CEN2* and loss of telomere adjacent sequences, could have resulted from a closed fork distal to *SUL1*.

The frequency with which we recover inverted *SUL1* amplicons is probably not due to structural or sequence properties of the chromosomal domain that contains *SUL1*: The density of small, interrupted inverted repeats surrounding the *SUL1* locus is similar to that found in other regions of the genome—roughly one such sequence (7 bp inverted repeats with spacing of less than 70 bp) every 240 bp. Instead, the reason that *SUL1* amplification is so common is likely due to the selective advantage conferred by the extra copies of *SUL1* for cells growing in a sulfate-limited chemostat (a ~40% growth advantage over the ancestral strain; [4]). Moreover, even before the dimeric plasmid integrates to generate a more stably inherited amplicon, cells with the dimeric circle are expected to experience a similar selective advantage [4, 16].

In addition to the ODIRA model, there are two general classes of replication models that could explain the alternating inverted structure of the *SUL1* amplicons. One class includes MMBIR and FoSTeS [6]. The common feature of these models is that a free 3’ DNA end, from either a DNA break or a stalled replication fork, carries out a search for microhomology at which the free 3’ end can initiate replication by strand invasion. In FoSTeS, the 3’ end can undergo multiple sequential invasions to generate complex rearrangements. The second class of models that can generate Tandem Inverted Duplications (TIDs), described by Kugelberg et al. [24], suggests that replication stress causes a leading strand to fold back on itself at a pair of short interrupted inverted repeats, copying the newly synthesized nascent strand until a second pair of short interrupted inverted repeats is encountered, at which point synthesis of the template strand resumes. After the next round of replication, the newly synthesized strand contains a structure that is identical to the ones we find at the *SUL1* locus, but in this model for amplification there is no requirement for the presence of an origin of replication in the amplified segment.

Nevertheless, just because it is possible to draw a pleasing model on paper, doesn’t make it true. Substantiating the individual stages in the process is an important step in confirming that the model is plausible. Here we show that the initiating event of the ODIRA model—fork reversal at the site of short (7 bp) interrupted repeats allowing the leading strand 3’-end to migrate to the lagging strand and become ligated to the lagging strand—can create a closed fork. While we have not assayed the process of loop extrusion *in vitro*, the same process of branch migration that created the closed fork is expected to assist its removal from the two parental strands. *In vivo*, when a neighboring replication fork approaches the closed fork, the combination of helicases and topoisomerases traveling with the advancing fork are precisely situated to assist the removal of the closed fork to generate a dog bone linear structure with single stranded loops protecting the ends from degradation. Because the dog bone would persist only until the next S-phase, it is unlikely that we would ever be able to detect these molecules *in vivo*. However, we find that the artificial dog bone constructed from *URA3* and *ARS1* sequences transforms yeast at high frequency and is maintained after replication as a dimeric, inverted plasmid with junctions derived from the artificial hairpin adapters ligated on *in vitro*. From the high frequency of transformation we obtain with the artificial dog bone, we conclude that dog bone structures are not a target for degradation, but are efficiently replicated *in vivo* to create the inverted dimeric plasmid. Over a period of less than 100 generations, we can begin to detect integration into the *ARS1* locus on chromosome IV with the product being an inverted triplication of the *ARS1* fragment.

Recently the Regenberg lab reported that circular DNA derived from genomic DNA is quite common in yeast [25]. Their genome-wide scan for circles identified more than a thousand unique sequences, 80% of which contained a known yeast origin of replication. However, they did not find a circle containing *SUL1* and its adjacent origin, *ARS228*. Many of their recovered circles had arisen by homologous recombination between direct repeats: they did not report finding any circles with an inverted structure. It is not clear to us whether the DNA polymerase from Phi29 that they used for rolling circle amplification would have successfully amplified palindromic circles. In general, palindromic sequences are difficult to amplify and sequence because they do not remain single stranded after denaturation [26]. DNA samples that we have examined over the course of the sulfate limited chemostat growth have also not yet revealed the presence of a free inverted dimeric plasmid that are analogous to the *URA3-ARS1* plasmids in the current study. Together, these results suggest that if there is a circular *SUL1* intermediate, it is short lived and that stable integration is strongly selected for by growth in the limited sulfate medium. The difference in prevalence of the presumptive *SUL1* circular intermediate and the *URA3-ARS1* inverted dimer could also arise from the different nature of the selective pressures operating on *SUL1* and *URA3*. *SUL1* is dosage sensitive—cells with a single, extra copy of *SUL1* are less fit than cells with multiple extra copies of *SUL1* [4, 16]. *URA3* is not known to be dosage sensitive, as multiple copies provide no obvious growth advantage over a single copy. Alternatively, the *SUL1* locus, residing ~25 kb from the right telomere of chromosome II, may be in a more accessible portion of the genome for homologous recombination than *ARS1* which is located 13 kb from the centromere of chromosome IV.

A second difference that exists between the majority of naturally occurring inverted amplicons of *SUL1* and the artificial ones of the *URA3-ARS1* fragment is in the stability of the junctions. Among the two dozen *SUL1* amplicons we have characterized, we have only found two in which the centromere-proximal junction suffered an asymmetric deletion of one arm of the palindrome (Figs 8 and S4) [4]. In the five batch culture evolution experiments we performed on the *URA3-ARS1* dog bones, it is apparent that the plasmid changes over time (Figs 5 and S3) and that those changes include either the loss of some of the restriction sites included in the hairpin termini or loss of sequences from one of the palindromic arms. The reasons for this apparent difference in stability may be due to the size of the loops: the artificial loops are only 2–4 bases, while the smallest loop recovered from stable, inverted *SUL1* junctions is 17 bp (S4 Fig; [4]). It is likely that a greater separation of the two halves of a large palindrome would increase its stability, a phenomenon first discovered in plasmids in *E*. *coli* [27].

The two *SUL1* amplicons with secondary deletions provide insight into the generation of an increasingly recognized type of human triplication—the DUP-TRP/INV-DUP CNV where the central inverted copy contains only a subset of the sequences included in the directly repeated flanking duplicated segments [8]. By analyzing the sequences at the junctions of the two yeast amplicons, we have been able to recreate a plausible series of events that led to the recovered amplicon. Perhaps because yeast has so few dispersed, repeated sequences, and because the *SUL1* region, in particular, lacks any significant repetitive sequences, we have been able to detect an amplification process that is initiated by this heretofore unrecognized potential replication error. Using our *in vitro* and *in vivo* model systems we have been able to reconstruct the major events of the ODIRA model.

FoSTeS, MMBIR and Kugelberg’s model for the generation of TIDs can also explain how inverted *SUL1* amplicons arise. What ODIRA provides in addition to an explanation for the generation of DUP-TRP/INV-DUPs and TIDs is a role for the origin that lies at the 3’ end of *SUL1*. It is the presence of an origin of replication in all *SUL1* amplicons that we find intriguing. Additionally, the extrachromosomal intermediate in the ODIRA model provides a simple explanation for how some human *de novo* inverted triplications contain sequences from both homologues of one parent [22, 28, 29]. Taken together, our results on *SUL1* amplicons, artificial forks, dog bones and ARS deletions support the individual key steps of the ODIRA model for generating inverted triplication and provide approaches for further study of this fascinating and important form of genome evolution.

## Methods

### Plasmids and yeast strains

The plasmid pUA-DirB, constructed for other purposes, contains the *PflM*I-*Ava*I *URA3* fragment from Yip5 [30] cloned next to a *Hind*III-*EcoR*I fragment containing *ARS1* from pΔ4 [31] in a pUC18 backbone. The yeast strain used for transformation is BY4741 [32] containing a complete deletion of *URA3* coding sequences and the 5’ and 3’ flanking sequences contained on vector pUA-DirB. Cells were grown at 30°C in synthetic complete medium (C); transformants were grown in C medium lacking uracil (C-uracil). Sulfate-limited chemostat growth was performed as described on strains with the S288C background [16, 18]. Individual clones (S10206-C1 and S20107-C1) were selected after ~200 generations in the chemostats and characterized as previously described [4]. The method for whole genome sequencing and SplitReads analysis were performed previously as described [4]. The clone, S20107-C1, was included in a previous analysis (Pop7 clone1, [4]), the raw data for clone S10206-C1 are deposited at BioProject ID PRJNA291644 and BioSample numbers SAMN03951108. Microarray data from this article have been deposited in the Gene Expression Omnibus repository under accessions GSE47854, GSE74443, GSE36691 and GSE13435 (http://www.ncbi.nlm.nih.gov/geo/) and in the Princeton Microarray Database (http://puma.princeton.edu).

We removed origin function of *ARS228* by shuffling and reverse complementing the bases within the ARS consensus sequence, changing AACAAAACATAATTTC to TAATAGTGATGTTATA using PCR fusion (primers listed in S1 Table; shuffled ARS ACS and *Bgl*II sequences are in lower case) and a pop-in/pop-out strategy [33]. The *Bgl*II fragment from the fused PCR product was cloned into pRS306, sequenced, linearized with *Hpa*I and transformed into a *ura3* strain of S288C. After selecting for uracil prototrophy, we selected 5FOA resistant colonies and confirmed the *ARS228* mutation by Sanger sequencing (primers *ARS228-for* and *ARS228-rev*). We confirmed loss of origin function by 2D gel electrophoresis (S6 Fig) [13].

### Nucleic acids and gel analysis

Custom oligonucleotides for the artificial fork, listed in S1 Table, were purchased from Invitrogen or Integrated DNA Technologies, resuspended in 10 mM Tris (pH 8), 0.1 mM EDTA, and kept frozen at -20°C. To form the artificial replication fork, equimolar amounts of the leading and lagging oligonucleotides were mixed at a final concentration of ~65 ng/μl (~0.15 μM) each in buffer #3 (NEB), heated for 15 minutes at 100°C, centrifuged briefly to collect condensation, and plunged into an ice bath for 10 minutes before being returned to 23°C. T4 DNA ligase and ATP were added and the mixture incubated at 23°C for 10–30 minutes. Samples were analyzed by electrophoresis on 3.75% GTG agarose (Lonza) gels with 1X Tris-borate EDTA (TBE) and 0.3 μg/ml ethidium bromide at ~4 volts/cm for 45–60 minutes. PCR analysis of the ligated fork was performed with AmpliTaq polymerase (Invitrogen) as follows: 3 min at 95°C, 30 cycles of 30 sec at 95°C, 30 sec at 55°C and 30 sec at 72°C, followed by 1 min at 72°C.

The *URA3-ARS1* dog bone was constructed by ligating the 2.3 kb *Hind*III-*Xba*I fragment of pUA-DirB with the *Hind*III and *Xba*I hairpin molecules at a molar ratio of 1:60:60 with T4 DNA ligase at 23°C for 1 hour. The strain BY4741 was transformed with the lithium acetate procedure [34] and transformants selected on C–uracil plates. Clones were colony purified and DNA preps made from an overnight stationary phase culture grown in liquid C–uracil medium. Serial passage of transformants in C-uracil medium was conducted by inoculating 5 ml of fresh medium every second day with 5 μl of saturated culture for a total of 8 or 9 passages, constituting ~100 generations of selective growth. A single clone was selected from each final culture by plating on C-uracil medium. “Smash-and grab” [35] DNA was used for restriction enzyme digests, snap-back assays [4], and PCR analysis of the *URA3-ARS1* dog bones, and aCGH analysis of the two *SUL1* amplified clones, S20106 C1 and S10207 C1. Restriction digests and snap-back assays on samples S20106 C1 and S10207 C1, were performed on high molecular weight DNA isolated by the Nib-n-grab protocol [4] and electrophoresed on 0.4% agarose gels at 1 V/cm for 16–20 hrs. Restriction enzymes were purchase from New England Biolabs; S1 nuclease was purchased from Promega. Plug samples were used for CHEF gel analysis [4] run under conditions to separate chromosomes IV and XII: 1% LE agarose gel in 0.5X TBE at 14°C for 68 hours at 100 volts with switch times ramped from 300 seconds to 900 seconds using a BioRad DRII CHEF gel apparatus.

2D gel electrophoresis to detect replication initiation at *ARS228* was performed according to [13]. To assess the consequence of the scrambled *ars228D* sequence on origin function, genomic DNA (6 μg) from log phase cultures of FY3 and FY3/*ars228Δ* cells was cleaved with *Bgl*II to release a 3.85 fragment with *ARS228* near its center. The Southern blot was probed with an *ARS228* probe. To determine the functional status of *ARS228* and *ARS228*.*5* in clones where the inversion junctions were in close proximity to the ARSs, DNA from exponentially growing cultures of clones Pop5c1 and 10205-c1464 was digested with *Bcl*II or *Afl*II, respectively, and probed with fragments from *SUL1* or *PCA1*, respectively. These digests distinguish between the ARS sequence residing in the ancestral fragment from the unique fragment spanning the inversion junctions.

PCR products from *SUL1*, *ARS228*, *PCA1*, *URA3* and the region adjacent to *ARS1* were labeled with α-^32^P-dATP by random priming using exo-minus Klenow polymerase (New England Biolabs). Southern blotting and hybridization were carried out according to standard procedures.

## Supporting Information

S1 Table Custom oligonucleotides. (DOCX)

S1 Movie A two-dimensional animation of fork migration at short inverted repeats. The leading and lagging oligos (S1 Table) are drawn with the fold-back duplexes that create the nascent arms of the replication fork. The two oligos are held together by a 30 bp stretch of complementarity, reflecting the unreplicated parental duplex at a replication fork. The inverted repeats (in blue) lie in the single stranded Okazaki gap on the lagging strand. The animation steps through 25 steps of replication fork regression and illustrates the intermediates in which the 3’ C (in red) of the leading strand comes to lie next to the 5’T (in red) on the lagging, using the complementarity of the inverted repeats (in blue) as a template for annealing. There are 31 frames, numbered from 100 to 130, with branch migration beginning in frame 104.
(MOV)

S1 Fig Characterization of the 78 bp fork junction PCR product. The ligated, annealed leading and lagging oligos were used as a template for PCR as described in Fig 3B and subjected to further enzymatic treatments, including S1 nuclease, *Hae*III and *Nco*I which cleave in the parental duplex portion of the annealed oligos, and *Xho*I which only can cleave a successfully ligated fragment that joins the leading and lagging nascent strands.
(TIF)

S2 Fig Confirmation of the dogbone structure used to transform yeast. (A) Map of the ~2.3 kb *URA3-ARS1* fragment from pUA-DirB. The hairpins ligated at the *Hind*III and *Xba*I sites are not drawn to scale. The expected *Bgl*II and *Nco*I fragments are indicated in bp. (B) Ethidium bromide stained gel of the *URA3-ARS1* fragment with and without ligated hairpins and with and without *Bgl*II or *Nco*I. Notice that ligation of the hairpins causes the fragment to migrate at a slightly larger size (compare first two lanes) and that this up-shift in size is detected on each end (compare middle two lanes and last two lanes).
(TIF)

S3 Fig Loss of DNA from the *URA-ARS1* inverted dimeric plasmids on long-term growth. Genomic DNA isolated from the first day population samples and the last day clones for transformants #1 and #5 were analyzed as described in Fig 4. The gray shaded sections over the plasmid maps indicate the restriction sites that were present in the initial culture but missing from the last day clone. (A) Treatment with *Xho*I and *Hind*III linearize the plasmids from the first day sample, but leave the plasmid from the last day clone uncut (SC = supercoils; NC = nicked circles). The loss of DNA sequence from the *URA3*-adjacent hairpin is also evident by comparing the sizes of restriction fragments between the first and last samples: red dotted lines placed over the bands of the first day samples highlight the restriction fragments that are shorter by ~150 bp in the last day clone. (B) Comparison of the uncut samples from the first and last day samples of transformant #5 indicates a large reduction in plasmid size. Cleavage of the plasmid with each enzyme in turn generates an identically sized linear fragment consistent with loss of an entire arm of the palindromic plasmid. However, it is also evident that the restriction sites included in the two hairpins are still present in the deleted plasmid.
(TIF)

S4 Fig Secondary rearrangement at a palindromic junction in a *SUL1* amplicon. (A) aCGH of yeast strain Pop7-c1. Only the last 65 kb of chromosome II are shown. (B) Sequences of the ancestral chromosome and the rearranged palindromic junction from the evolved clone [4]. Sequences centromere-proximal of the inversion junction are in lower case. The short inverted repeats proposed to be the site of fork closure are indicated in red font (red arrow); sequences believed to be the sites of homologous pop-out recombination to generate the deletion of one arm of the palindrome are underlined (black arrow). Colored highlights are included to illustrate the orientation and junctions created after formation of the amplicon and the deletion of one of the palindromic arms. (C) Map illustrating the structure of the original inferred inverted triplication, highlighting the short inverted repeats (red arrows) and the site of homologous recombination (black arrows). Colored blocks are the same as in (B). The direct orientation of the two distal black arrows creates an opportunity for pop-out recombination to remove the intervening sequences. (D) Confirmation of the structure of the centromere-proximal amplicon junction by indirect end-labeling. DNA from Pop7-c1 was digested with *EcoN*I, distributed to 9 tubes and digested with one of the indicated enzymes. A probe adjacent to the *EcoN*I site detects fragments that extend from the *EcoN*I site toward the centromere. The map in (C) illustrates the locations of sites in the ancestral genome; these fragment sizes are detected for the most centromere proximal copy of the *SUL1* locus and for fragments completely contained within the two arms of the inverted amplicon. The deletion lies between the *Apa*I and *Kpn*I sites, with a single *Afl*II site remaining in the un-deleted arm of the palindrome. (E) Snap-back assays on *EcoN*I and *ApaL*I digested genomic DNA from Pop7-c1. Both ancestral and amplicon specific *EcoN*I fragments are detected in native DNA. Denaturation and quick cooling produces a smear of ssDNA fragments along with a duplex hairpin of approximately 13 kb. An 11 kb duplex portion of the hairpin was resistant to S1 nuclease digestion. The reduction in size is consistent with the hairpin having lost a loop of approximately 2000 nucleotides during S1 digestion. Ancestral and amplicon specific *ApaL*I fragments are not resolved for native DNA. Denaturation and quick cooling produces a similar smear of ssDNA fragments with a duplex hairpin of approximately 13 kb that does not change appreciably after S1 nuclease digestion.
(TIF)

S5 Fig Molecular characterization of amplicons using the snap-back/S1 assay. *ApaL*I digests of DNA from evolved clones reveals duplex fragment that are resistant to S1 nuclease after denaturation and quick-cooling (snap-back). Among the original clones from the seven *ars228Δ* chemostats, (A) four clones contained inverted amplicons, and (B) three proved to have structures consistent with tandem addition (or translocation) of *SUL1* telomere fragments to chromosome II or other chromosomes. In the Southern blots, a blue bar marks the position of the expected ancestral terminal *ApaL*I fragment; red bars mark the variable positions of the amplified *SUL1 ApaL*I fragments. After denaturation and S1 treatment the inverted amplified fragment is reduced in size and resistant to S1 treatment (red angled arrows). The absence of the S1 protected band in the DNA from clones in (B) is consistent with tandem direct or translocated amplification events.
(TIF)

S6 Fig 2D gel analysis of *ARS228* origin function. The *Bgl*II fragments from *ARS228* (A) and *ars228Δ* (B) strains were detected by hybridizing the Southern blot of the 2D gel with an *ARS228* probe. Replication bubbles (red arrow), indicative of an active origin within the fragment, are only detected in the strain with wild type *ARS228*. The replication intermediates for *ars228Δ* are simple-Ys (black arrow), indicative of passive replication through the fragment by forks from adjacent origins.
(TIF)

S7 Fig 2D gel analysis of *ARS228* and *ARS228*.*5* origin function in amplified clones. Two evolved clones with a single ARS element in close proximity to the inversion junction were analyzed for origin function. Clone Pop5 c1 includes *ARS228* and 10205-c1464, derived from the ars*228Δ* strain, contains *ARS228*.*5*. Digestion of Pop5 c1 genomic DNA with *Bcl*II (A, C, and E) generates an ancestral fragment (blue) that is smaller than the fragment containing the inversion junction (red), while digestion of 10205-c1464 genomic DNA (B, D, and E) generates an ancestral fragment (blue) that is larger than the inversion junction fragment (red). The inversion fragments in both clones produce similar sized fragments and both display prominent bubble arcs (red arrows) indicating robust origin function, despite their new contexts. (Because of its large size, the replication intermediates from the ancestral fragment from clone 10205-c1464 does not resolve into distinct bubble and Y species.)
(TIF)
