# Supplementary material for: Origin-Dependent Inverted-Repeat Amplification: Tests of a Model for Inverted DNA Amplification
Source: PLoS Genet. 2015 Dec 23;11(12):e1005699. doi: 10.1371/journal.pgen.1005699 (PMC4689423; doi:10.1371/journal.pgen.1005699)
Supplement: S1 Table — (DOCX) [file pgen.1005699.s001.docx]

S1 Table. Custom Oligonucleotides

| Oligo | Sequence |
| --- | --- |
| Leading Strand | 5’ CGA ATG CCA TTA GAT CGG ACT TAG GCC ATG GAT AAA GAC CAG AAA ATC CTT TAT CTG CAG TGT AGA CGC ACT GCA GTA GAT AAA GGA TTT TCT GGT CTT TAT C 3’ |
| Lagging Strand | 5’-phosphate TCG AGG CTC AGG GTA CCT GCG TTA CCA GGT ACC CTG AGC CTC GAG ATA AAG GAT TTT CTG GTC TTT ATC CAT GGC CTA AGT CCG ATC TAA TGG CAT TCG 3’ |
| Leading Primer | 5’ TGC AGT GTA GAC G 3’ |
| Lagging Primer | 5’ GGT ACC TGG TAA CG 3’ |
| *Hind*III hairpin | 5’-phosphate AGC TTG GCT ACT GCT CGA GTA GGT TAA CTA ACC TAC TCG AGC AGT AGC CA 3’ |
| *Xba*I hairpin | 5’-phosphate CTA GAT ACT GGT ACG TAT GGT TAA CAC CAT ACG TAC CAG TAT 3’ |
| *URA3* Primer | 5’ TAG ACC CGT GGA TGA TGT GG 3’ |
| *GAL3* Primer | 5’ TGC CTC ACT TGT CGC TTA TG 3’ |
| *ARS228*ACSdelF_5 | 5’ ATA AAT TAa gat ctA TGA AAA GCC GCT ACT GAA AGT CGT C 3’ |
| *ARS228*ACSdelR_6 | 5’ taa tag tga tgt tat aTT CCA CAT TAG GAG AGA CAA GC 3’ |
| *ARS228*ACSdelF_6 | 5’ tat aac atc act att aAT GAA TCT GAA AAG AAA AAT ACT ATT TTA GC 3’ |
| *ARS228*ACSdelR_5 | 5’ ATA AAT AAa gat ctA AAC ATC CAG AAC TGT TGC GGT AG 3’ |
| *ARS228-for* | 5’ GCT TAT GCT AGA TAG AGC AAC ATT TTC C 3’ |
| *ARS228-rev* | 5’ AAC AGT GAT GGA TTC ATC CGC TCG 3’ |
