# Supplementary figures and images for: Origin-Dependent Inverted-Repeat Amplification: Tests of a Model for Inverted DNA Amplification
Source: PLoS Genet. 2015 Dec 23;11(12):e1005699. doi: 10.1371/journal.pgen.1005699 (PMC4689423; doi:10.1371/journal.pgen.1005699)

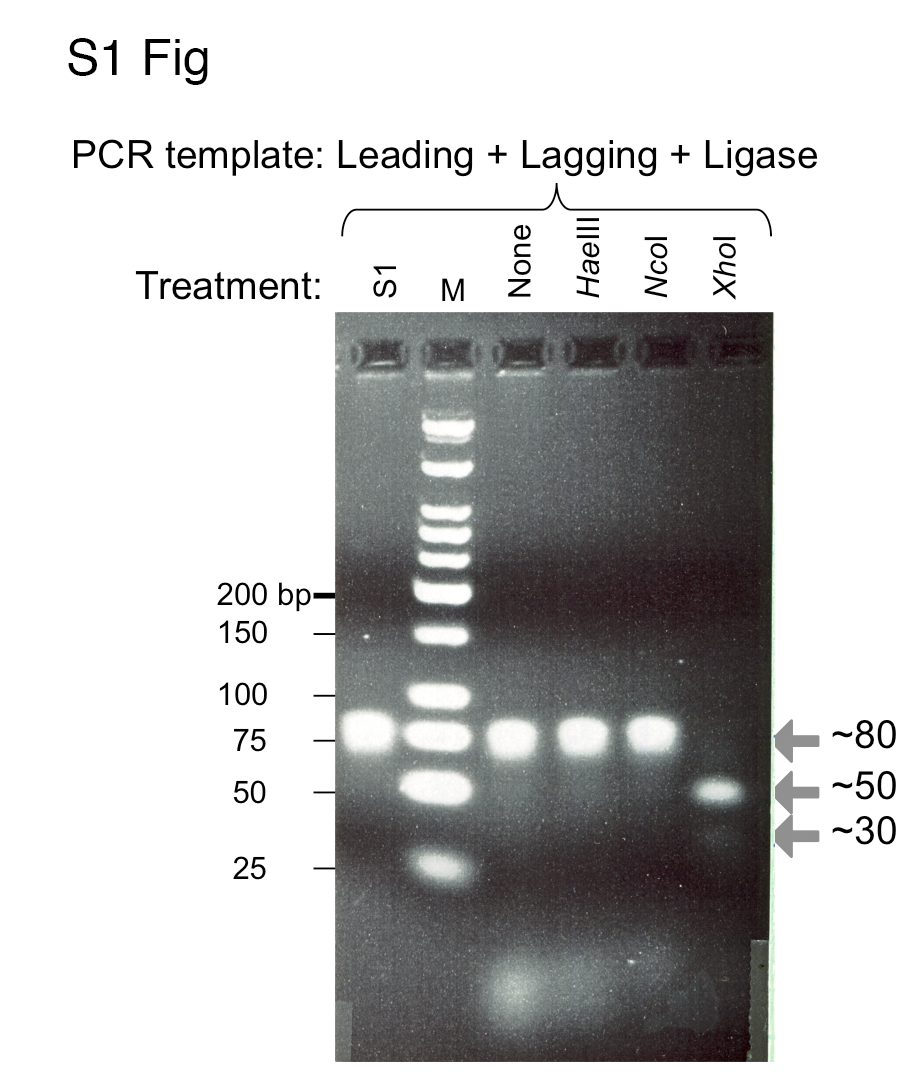

Supplement: S1 Fig — The ligated, annealed leading and lagging oligos were used as a template for PCR as described in Fig 3B and subjected to further enzymatic treatments, including S1 nuclease, HaeIII and NcoI which cleave in the parental duplex portion of the annealed oligos, and XhoI which only can cleave a successfully ligated fragment that joins the leading and lagging nascent strands. (TIF) [file pgen.1005699.s003.tif]

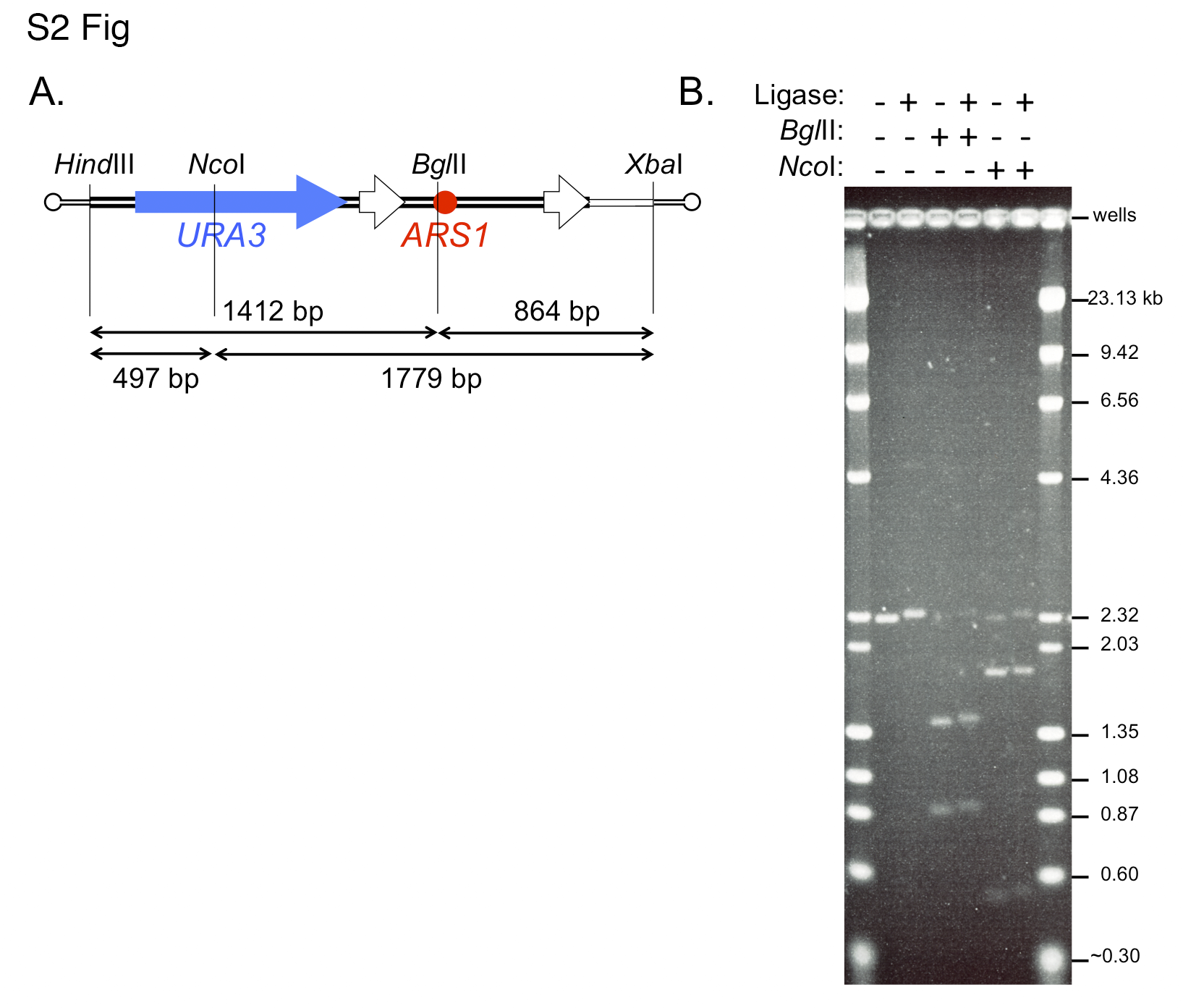

Supplement: S2 Fig — (A) Map of the ~2.3 kb URA3-ARS1 fragment from pUA-DirB. The hairpins ligated at the HindIII and XbaI sites are not drawn to scale. The expected BglII and NcoI fragments are indicated in bp. (B) Ethidium bromide stained gel of the URA3-ARS1 fragment with and without ligated hairpins and with and without BglII or NcoI. Notice that ligation of the hairpins causes the fragment to migrate at a slightly larger size (compare first two lanes) and that this up-shift in size is detected on each end (compare middle two lanes and last two lanes). (TIF) [file pgen.1005699.s004.tif]

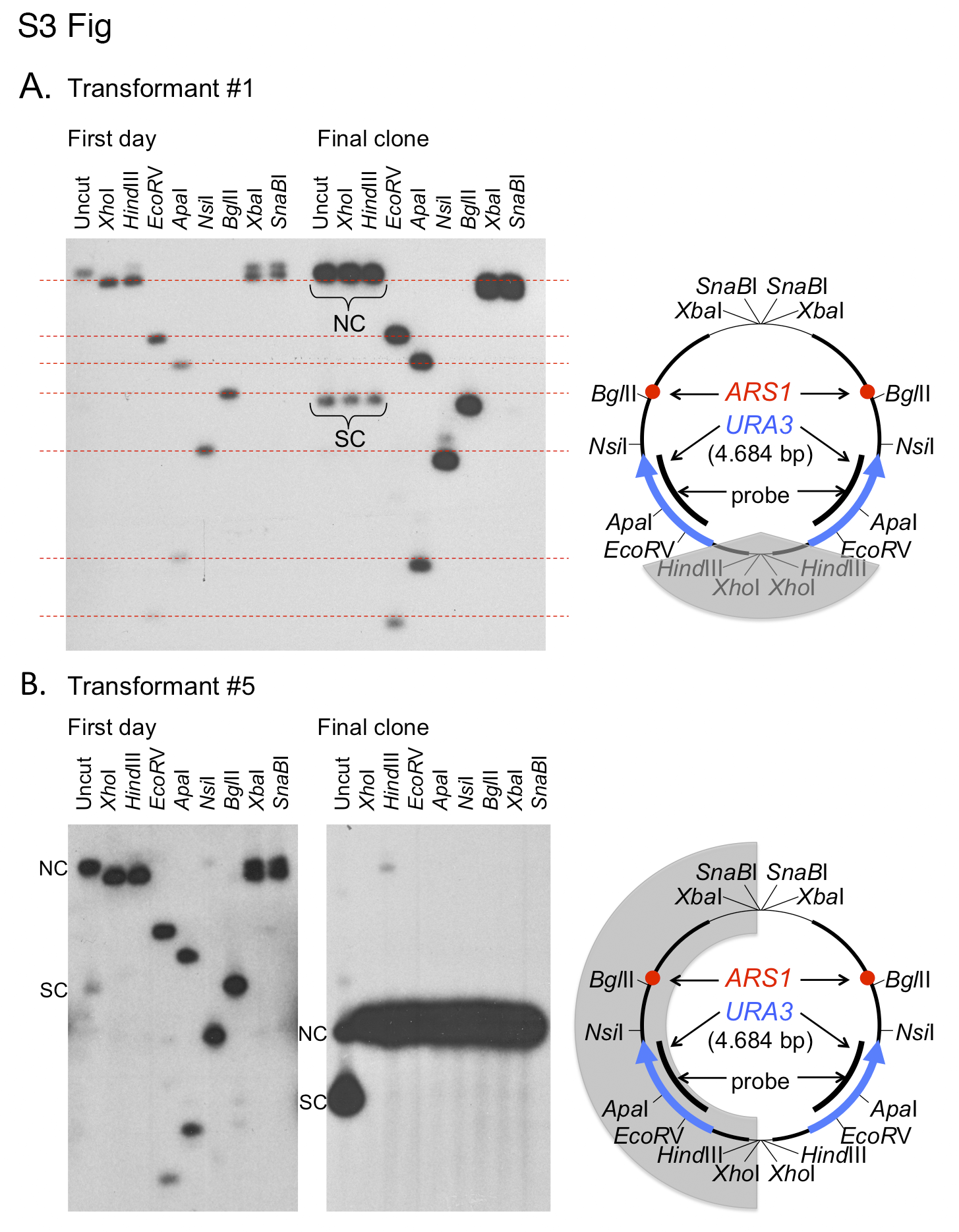

Supplement: S3 Fig — Genomic DNA isolated from the first day population samples and the last day clones for transformants #1 and #5 were analyzed as described in Fig 4. The gray shaded sections over the plasmid maps indicate the restriction sites that were present in the initial culture but missing from the last day clone. (A) Treatment with XhoI and HindIII linearize the plasmids from the first day sample, but leave the plasmid from the last day clone uncut (SC = supercoils; NC = nicked circles). The loss of DNA sequence from the URA3-adjacent hairpin is also evident by comparing the sizes of restriction fragments between the first and last samples: red dotted lines placed over the bands of the first day samples highlight the restriction fragments that are shorter by ~150 bp in the last day clone. (B) Comparison of the uncut samples from the first and last day samples of transformant #5 indicates a large reduction in plasmid size. Cleavage of the plasmid with each enzyme in turn generates an identically sized linear fragment consistent with loss of an entire arm of the palindromic plasmid. However, it is also evident that the restriction sites included in the two hairpins are still present in the deleted plasmid. (TIF) [file pgen.1005699.s005.tif]

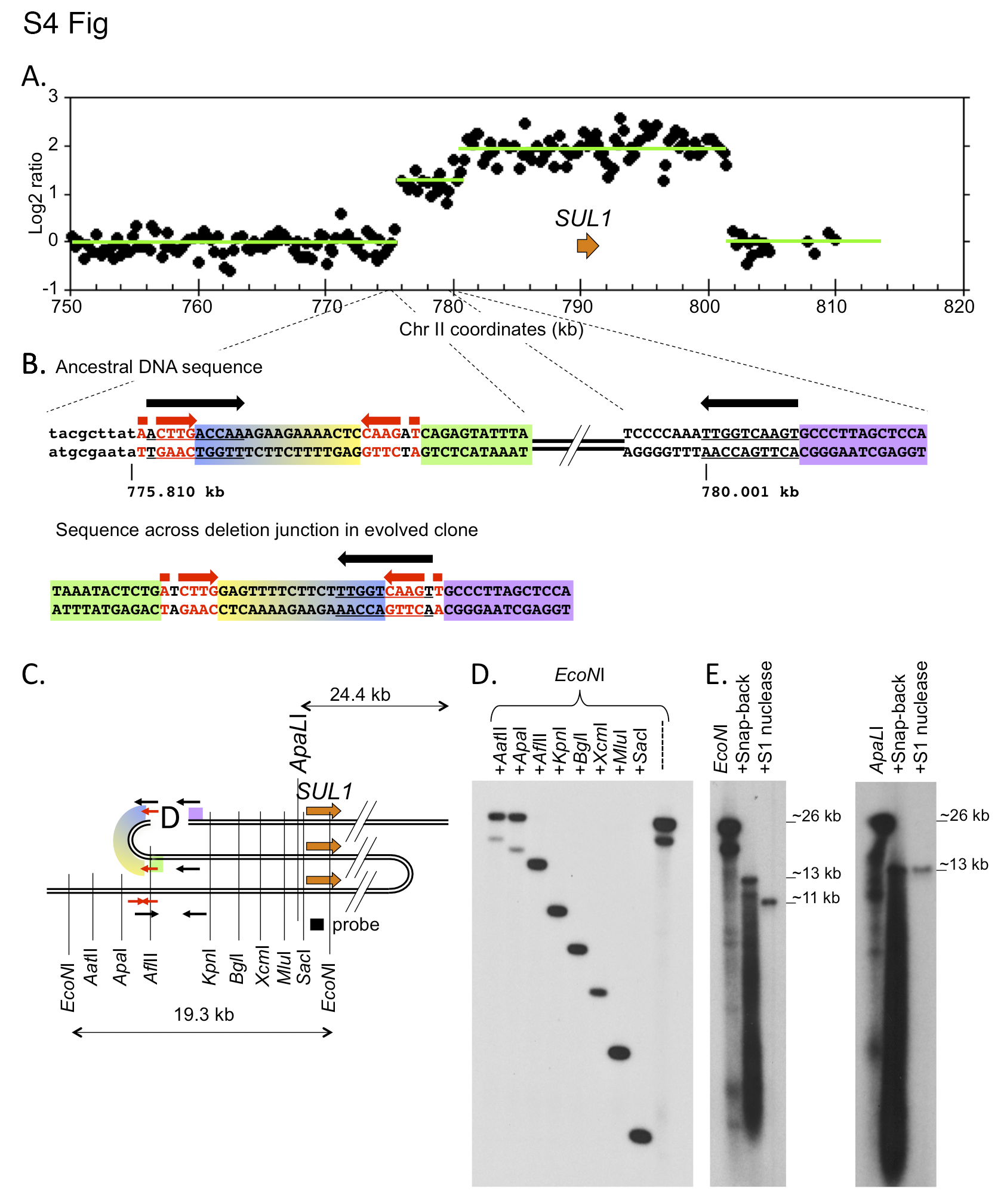

Supplement: S4 Fig — (A) aCGH of yeast strain Pop7-c1. Only the last 65 kb of chromosome II are shown. (B) Sequences of the ancestral chromosome and the rearranged palindromic junction from the evolved clone [4]. Sequences centromere-proximal of the inversion junction are in lower case. The short inverted repeats proposed to be the site of fork closure are indicated in red font (red arrow); sequences believed to be the sites of homologous pop-out recombination to generate the deletion of one arm of the palindrome are underlined (black arrow). Colored highlights are included to illustrate the orientation and junctions created after formation of the amplicon and the deletion of one of the palindromic arms. (C) Map illustrating the structure of the original inferred inverted triplication, highlighting the short inverted repeats (red arrows) and the site of homologous recombination (black arrows). Colored blocks are the same as in (B). The direct orientation of the two distal black arrows creates an opportunity for pop-out recombination to remove the intervening sequences. (D) Confirmation of the structure of the centromere-proximal amplicon junction by indirect end-labeling. DNA from Pop7-c1 was digested with EcoNI, distributed to 9 tubes and digested with one of the indicated enzymes. A probe adjacent to the EcoNI site detects fragments that extend from the EcoNI site toward the centromere. The map in (C) illustrates the locations of sites in the ancestral genome; these fragment sizes are detected for the most centromere proximal copy of the SUL1 locus and for fragments completely contained within the two arms of the inverted amplicon. The deletion lies between the ApaI and KpnI sites, with a single AflII site remaining in the un-deleted arm of the palindrome. (E) Snap-back assays on EcoNI and ApaLI digested genomic DNA from Pop7-c1. Both ancestral and amplicon specific EcoNI fragments are detected in native DNA. Denaturation and quick cooling produces a smear of ssDNA fragments along wit [file pgen.1005699.s006.tif]

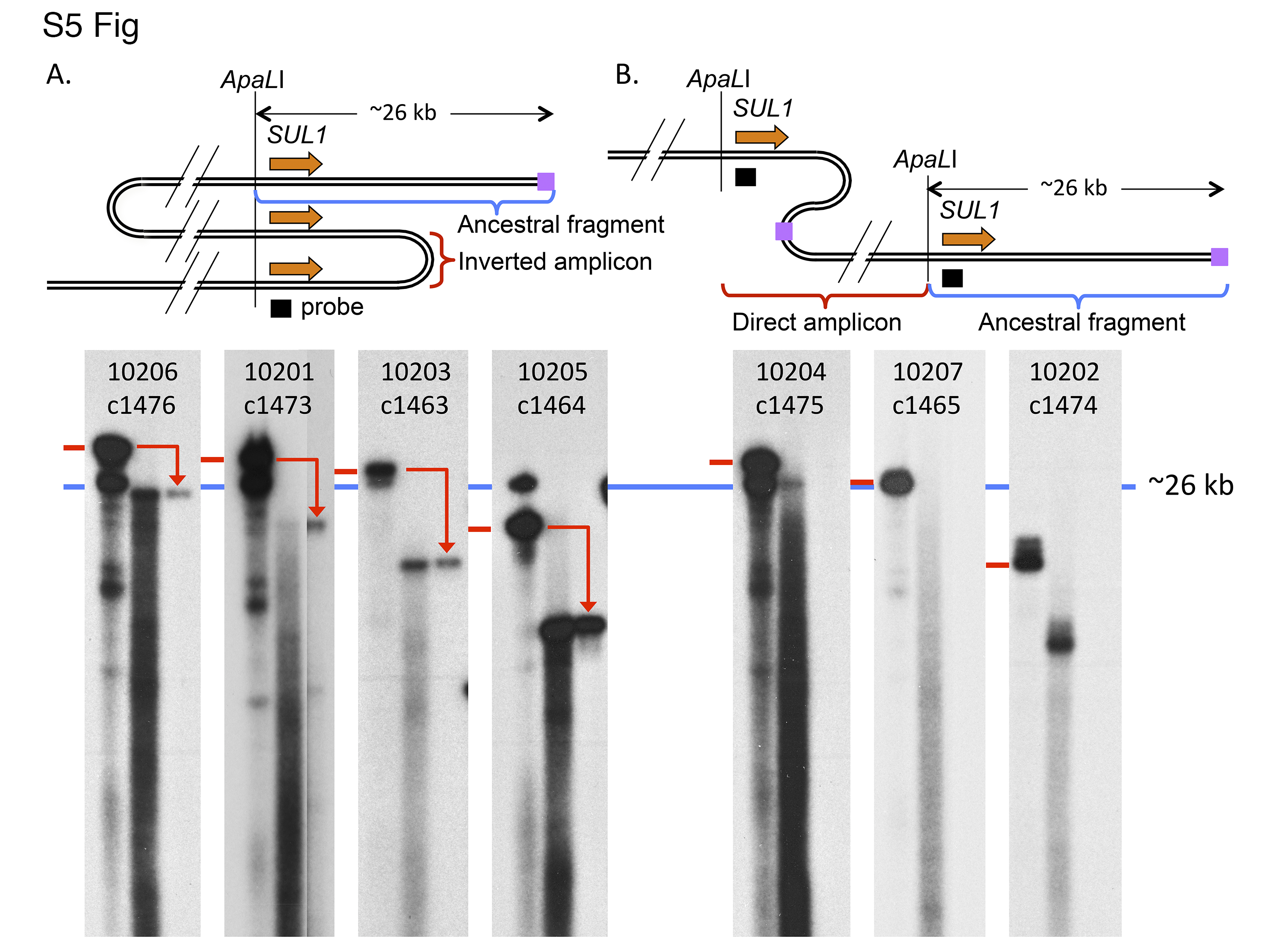

Supplement: S5 Fig — ApaLI digests of DNA from evolved clones reveals duplex fragment that are resistant to S1 nuclease after denaturation and quick-cooling (snap-back). Among the original clones from the seven ars228Δ chemostats, (A) four clones contained inverted amplicons, and (B) three proved to have structures consistent with tandem addition (or translocation) of SUL1 telomere fragments to chromosome II or other chromosomes. In the Southern blots, a blue bar marks the position of the expected ancestral terminal ApaLI fragment; red bars mark the variable positions of the amplified SUL1 ApaLI fragments. After denaturation and S1 treatment the inverted amplified fragment is reduced in size and resistant to S1 treatment (red angled arrows). The absence of the S1 protected band in the DNA from clones in (B) is consistent with tandem direct or translocated amplification events. (TIF) [file pgen.1005699.s007.tif]

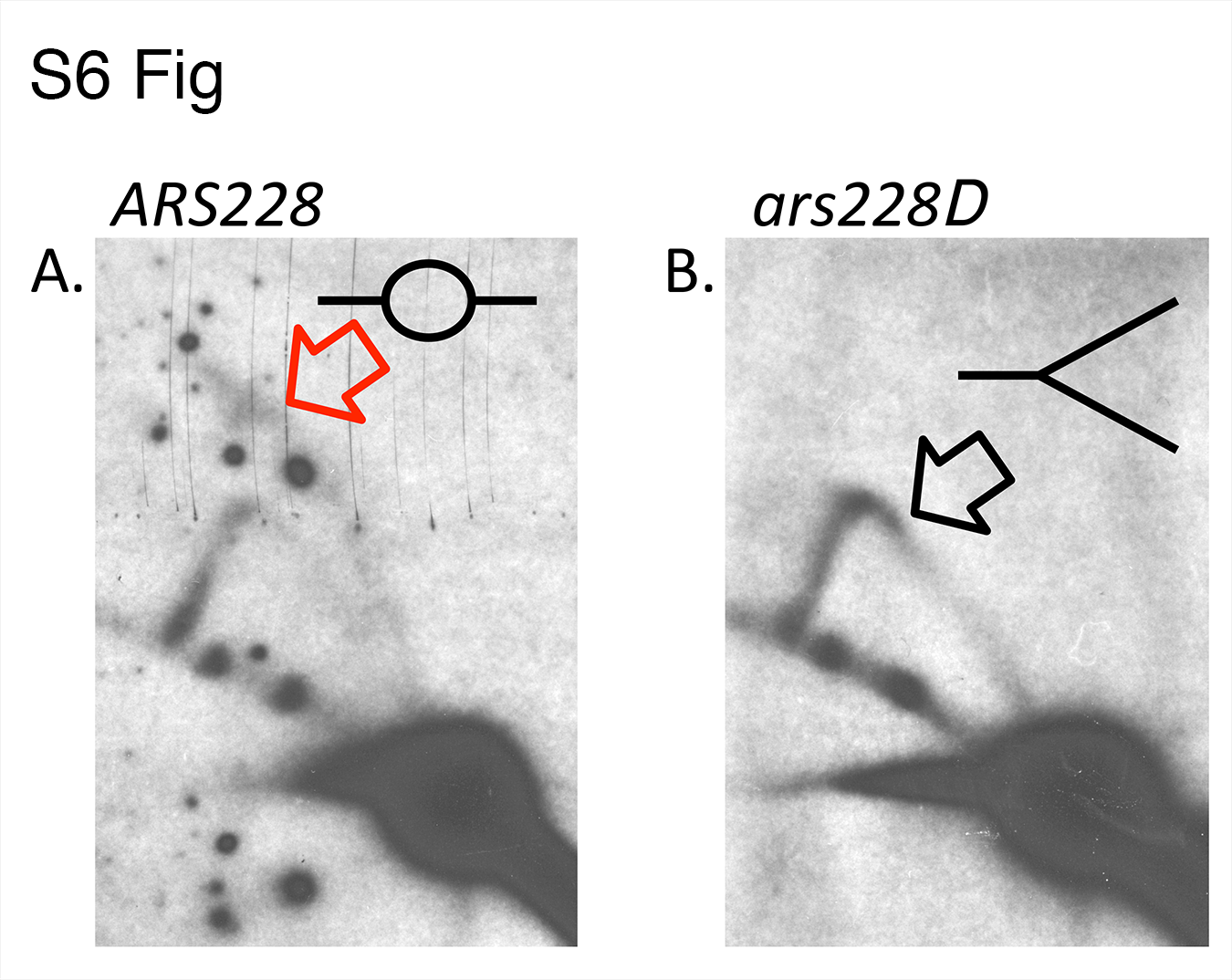

Supplement: S6 Fig — The BglII fragments from ARS228 (A) and ars228Δ (B) strains were detected by hybridizing the Southern blot of the 2D gel with an ARS228 probe. Replication bubbles (red arrow), indicative of an active origin within the fragment, are only detected in the strain with wild type ARS228. The replication intermediates for ars228Δ are simple-Ys (black arrow), indicative of passive replication through the fragment by forks from adjacent origins. (TIF) [file pgen.1005699.s008.tif]

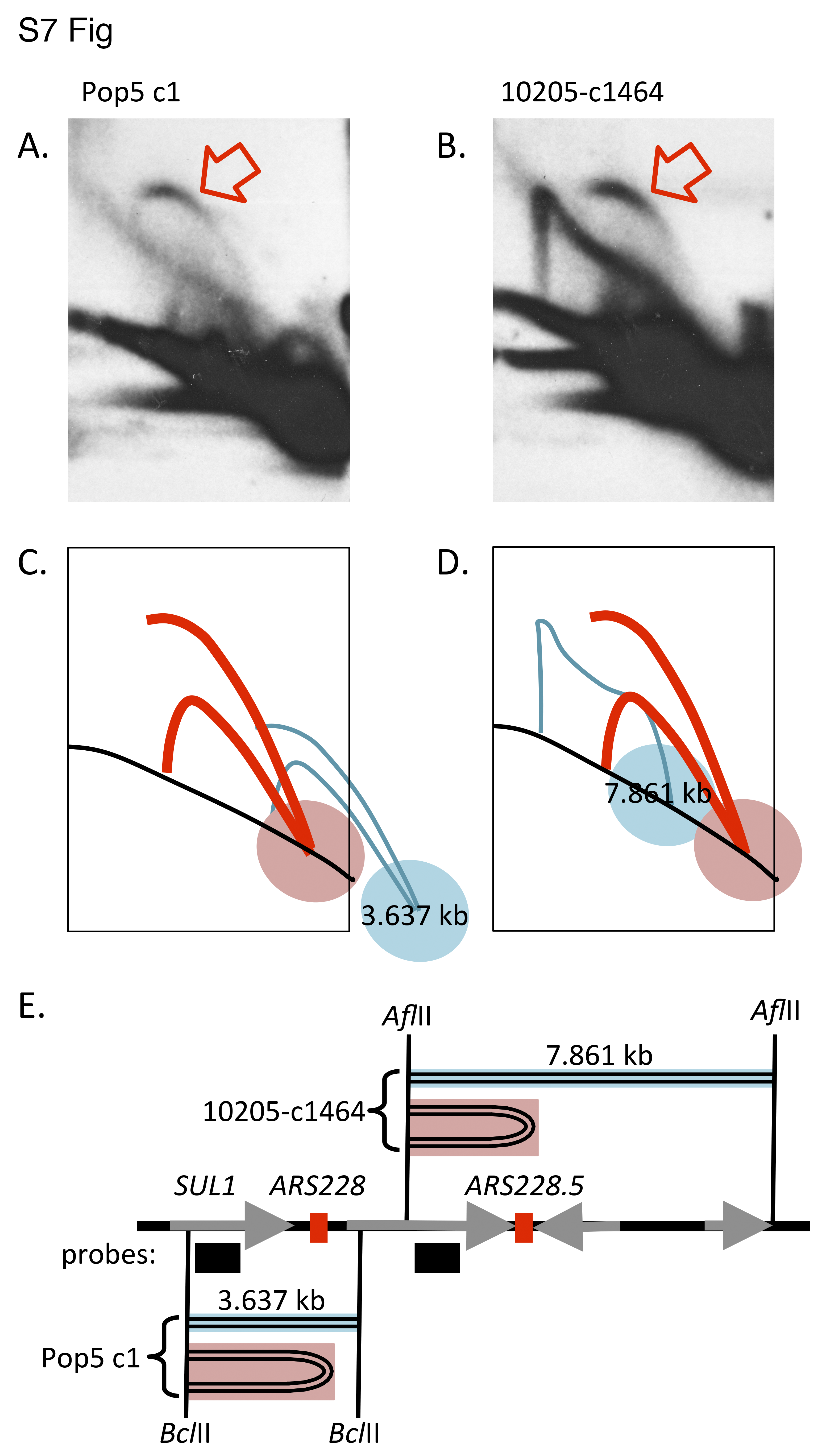

Supplement: S7 Fig — Two evolved clones with a single ARS element in close proximity to the inversion junction were analyzed for origin function. Clone Pop5 c1 includes ARS228 and 10205-c1464, derived from the ars228Δ strain, contains ARS228.5. Digestion of Pop5 c1 genomic DNA with BclII (A, C, and E) generates an ancestral fragment (blue) that is smaller than the fragment containing the inversion junction (red), while digestion of 10205-c1464 genomic DNA (B, D, and E) generates an ancestral fragment (blue) that is larger than the inversion junction fragment (red). The inversion fragments in both clones produce similar sized fragments and both display prominent bubble arcs (red arrows) indicating robust origin function, despite their new contexts. (Because of its large size, the replication intermediates from the ancestral fragment from clone 10205-c1464 does not resolve into distinct bubble and Y species.) (TIF) [file pgen.1005699.s009.tif]
